# Early diagnosis is associated with improved clinical outcomes in benign esophageal perforation: an individual patient data meta-analysis

**DOI:** 10.1007/s00464-020-07806-y

**Published:** 2020-07-17

**Authors:** Bram D. Vermeulen, Britt van der Leeden, Jawad T. Ali, Tomas Gudbjartsson, Michael Hermansson, Donald E. Low, Douglas G. Adler, Abraham J. Botha, Xavier B. D’Journo, Atila Eroglu, Lorenzo E. Ferri, Christoph Gubler, Jan Willem Haveman, Lileswar Kaman, Richard A. Kozarek, Simon Law, Gunnar Loske, Joerg Lindenmann, Jung-Hoon Park, J. David Richardson, Paulina Salminen, Ho-Yong Song, Jon A. Søreide, Manon C. W. Spaander, Jeffrey N. Tarascio, Jon A. Tsai, Tim Vanuytsel, Camiel Rosman, Peter D. Siersema, the Benign Esophageal Perforation Collaborative Group, Ruben D. van der Bogt, Madeleine Birch, Joseph J. Dubose, Sam Fox, Michael T. Jaklitsch, Madhan K. Kuppusamy, Saga Persson, Robert D. Rice, Josef Smolle, Freyja M. Smolle-Juettner, Monisha Sudarshan, Robert P. Sutcliffe, Halla Vidarsdottir, Asgaut Viste

**Affiliations:** 1 Department of Gastroenterology and Hepatology, Radboud University Medical Center, Nijmegen, The Netherlands; 2 Department of General Surgery, University of Texas at Austin, Dell Medical School, Texas, USA; 3 Department of Cardiothoracic Surgery, Landspitali University Hospital, Reykjavik, Iceland; 4 Department of Upper GI Surgery, Skane University Hospital, Lund, Sweden; 5 Department of Thoracic Surgery and Thoracic Oncology, Virginia Mason Medical Center, Seattle, WA USA; 6 Department of Medicine, Gastroenterology and Hepatology, University of Utah School of Medicine, Salt Lake City, UT USA; 7 Department of General and GI Surgery, Guy’s & St Thomas’s Hospitals, London, UK; 8 Department of Thoracic Surgery, Aix-Marseille Université, North Hospital, Marseille, France; 9 Department of Thoracic Surgery, Medical Faculty, Ataturk University, Erzurum, Turkey; 10 Department of Surgery and Oncology, McGill University, Montreal General Hospital, Montreal, Canada; 11 Klinik für Gastroenterologie und Hepatologie, Universitäts Spital Zürich, Zurich, Switzerland; 12 Department of Surgery, University Medical Center Groningen, University of Groningen, Groningen, The Netherlands; 13 Digestive Disease Institute, Virginia Mason Medical Center, Seattle, USA; 14 Department of Surgery, University of Hong Kong, Pok Fu Lam, Hong Kong; 15 Department for General, Abdominal, Thoracic and Vascular Surgery, Katholisches Marienkrankenhaus Hamburg gGmbH, Hamburg, Germany; 16 Division of Thoracic Surgery and Hyperbaric Surgery, Medical University of Graz, Graz, Austria; 17 Department of Vascular and Interventional Radiology, Asan Medical Center, University of Ulsan College of Medicine, Seoul, Republic of Korea; 18 Department of Surgery, University of Louisville School of Medicine, Louisville, USA; 19 Division of Digestive Surgery and Urology, Turku University Hospital, Turku, Finland; 20 Department of Gastrointestinal Surgery, Stavanger University Hospital, Stavanger, Norway; 21 Department of Clinical Medicine, University of Bergen, Bergen, Norway; 22 Department of Gastroenterology & Hepatology, Erasmus MC University Medical Center, Rotterdam, The Netherlands; 23 Division of Thoracic Surgery, Brigham and Women’s Hospital, Boston, MA USA; 24 Division of Surgery, Karolinska Institutet, CLINTEC, Stockholm, Sweden; 25 Department of Chronic Diseases, Translational Research Center for Gastrointestinal, KU Leuven, Leuven, Belgium; 26 Department of Surgery, Radboud University Medical Center, Nijmegen, The Netherlands; 27 Department of Gastroenterology and Hepatology (Route 455), Radboud University Medical Center, Geert Grooteplein-Zuid 8, 6500 HB Nijmegen, The Netherlands

**Keywords:** Esophageal rupture, Individual patient data meta-analysis, Time of diagnosis

## Abstract

**Background:**

Time of diagnosis (TOD) of benign esophageal perforation is regarded as an important risk factor for clinical outcome, although convincing evidence is lacking. The aim of this study is to assess whether time between onset of perforation and diagnosis is associated with clinical outcome in patients with iatrogenic esophageal perforation (IEP) and Boerhaave’s syndrome (BS).

**Methods:**

We searched MEDLINE, Embase and Cochrane library through June 2018 to identify studies. Authors were invited to share individual patient data and a meta-analysis was performed (PROSPERO: CRD42018093473). Patients were subdivided in early (≤ 24 h) and late (> 24 h) TOD and compared with mixed effects multivariable analysis while adjusting age, gender, location of perforation, initial treatment and center. Primary outcome was overall mortality. Secondary outcomes were length of hospital stay, re-interventions and ICU admission.

**Results:**

Our meta-analysis included IPD of 25 studies including 576 patients with IEP and 384 with BS. In IEP, early TOD was not associated with overall mortality (8% vs. 13%, OR 2.1, 95% CI 0.8–5.1), but was associated with a 23% decrease in ICU admissions (46% vs. 69%, OR 3.0, 95% CI 1.2–7.2), a 22% decrease in re-interventions (23% vs. 45%, OR 2.8, 95% CI 1.2–6.7) and a 36% decrease in length of hospital stay (14 vs. 22 days, *p* < 0.001), compared with late TOD. In BS, no associations between TOD and outcomes were found. When combining IEP and BS, early TOD was associated with a 6% decrease in overall mortality (10% vs. 16%, OR 2.1, 95% CI 1.1–3.9), a 19% decrease in re-interventions (26% vs. 45%, OR 1.9, 95% CI 1.1–3.2) and a 35% decrease in mean length of hospital stay (16 vs. 22 days, *p* = 0.001), compared with late TOD.

**Conclusions:**

This individual patient data meta-analysis confirms the general opinion that an early (≤ 24 h) compared to a late diagnosis (> 24 h) in benign esophageal perforations, particularly in IEP, is associated with improved clinical outcome.

**Electronic supplementary material:**

The online version of this article (10.1007/s00464-020-07806-y) contains supplementary material, which is available to authorized users.

Esophageal perforation is characterized by transmural disruption of the esophagus that could lead to contamination of the surrounding tissue. The majority of underlying causes consists of iatrogenic esophageal perforations (IEP) and spontaneous esophageal perforations, also known as Boerhaave’s syndrome (BS) [1]. The incidence of IEP is rising due to the increase in invasive endoscopic esophageal interventions in clinical practice [2–4].

Initial management of esophageal perforation generally consists of either surgical or endoscopic treatment (including drainage) combined with fasting, enteral tube feeding and intravenous antibiotics. Patients with either IEP or BS are usually managed with similar therapeutic strategies [5]. Nonetheless, optimal treatment selection for individual patients with esophageal perforation remains a challenge in current practice and is largely based on expert opinion as supportive high-level confidence evidence from comparative studies is generally lacking. Despite a reduction in mortality rates during the past decades, esophageal perforation continues to be associated with severe adverse clinical outcome [6]. A pooled meta-analysis published in 2013 showed a mortality rate of 12% [7], while a nation-wide population-based study in England reported a 35% overall mortality rate in patients diagnosed with predominantly BS between 2010 and 2012 [6]. The latter study also identified older age, type of therapeutic management (i.e., endoscopy) and lower patient volume per hospital as risk factors for a worse outcome in patients with esophageal perforation. Furthermore, BS was associated with a higher overall mortality rate when compared with IEP. This may be related to the higher risk of diagnostic delay as a result of the out-of-hospital setting in which BS often occurs.

When exploring the nature of clinical presentation, numerous case series have investigated whether time between onset and diagnosis is associated with clinical outcomes in patients with esophageal perforation [5, 8–13]. As a result, authors of some studies have suggested that diagnosis within 24 h, the so-called ‘golden 24 h-rule’, is associated with improved outcome in patients with any type of esophageal perforation [5, 9, 13]. However, given the design, heterogeneity in types of perforation and relatively small sample size of these studies, no convincing evidence for an association between time of diagnosis of esophageal perforation and clinical outcome has been found.

In an effort to pool results from published case series, we performed an individual patient data meta-analysis (IPDMA). This study design allows to combine raw patient data from case series and to stratify outcomes by type of perforation, while correcting for confounders in a multivariable analysis [14]. Our aim was to assess whether time of diagnosis (TOD) was associated with clinical outcomes in patients with IEP and BS.

## Patients and methods

The study protocol (PROSPERO: CRD42018093473) for the IPDMA was designed by the core members (B.V., C.R., P.S.) and approved by all collaborating authors of the Esophageal Perforation Study Group. The PRISMA guidelines for IPDMA and the MINORS critical appraisal tool for non-randomized interventional studies were followed [15, 16].

### Search strategy and study selection

A systematic literature search was performed in the electronic databases MEDLINE, EMBASE and Cochrane Central Register of Controlled Trials (CENTRAL) until June 30, 2017, which was updated until June 30, 2018 during the comprehensive process of inviting corresponding authors and data acquisition. Combinations of the following search terms with synonyms for “esophageal perforation”, “treatment” and “clinical study” were used to identify relevant studies (Supplementary Table 2). Thereafter, two researchers (B.V. and B.L.) independently screened and selected all studies according to the per protocol defined inclusion criteria: (1) adult patients treated for IEP or BS; (2) study reported original data on clinical outcome; (3) publications in English, Dutch, German; and (4) study size of ≥ 5 patients with IEP and/or BS. Studies were excluded when: (1) study exclusively reported on other types of esophageal perforation (e.g., malignant, external traumatic or intra-operative); (2) no full-text version available; (3) outcome only published as congress abstract.

**Fig. 1:**
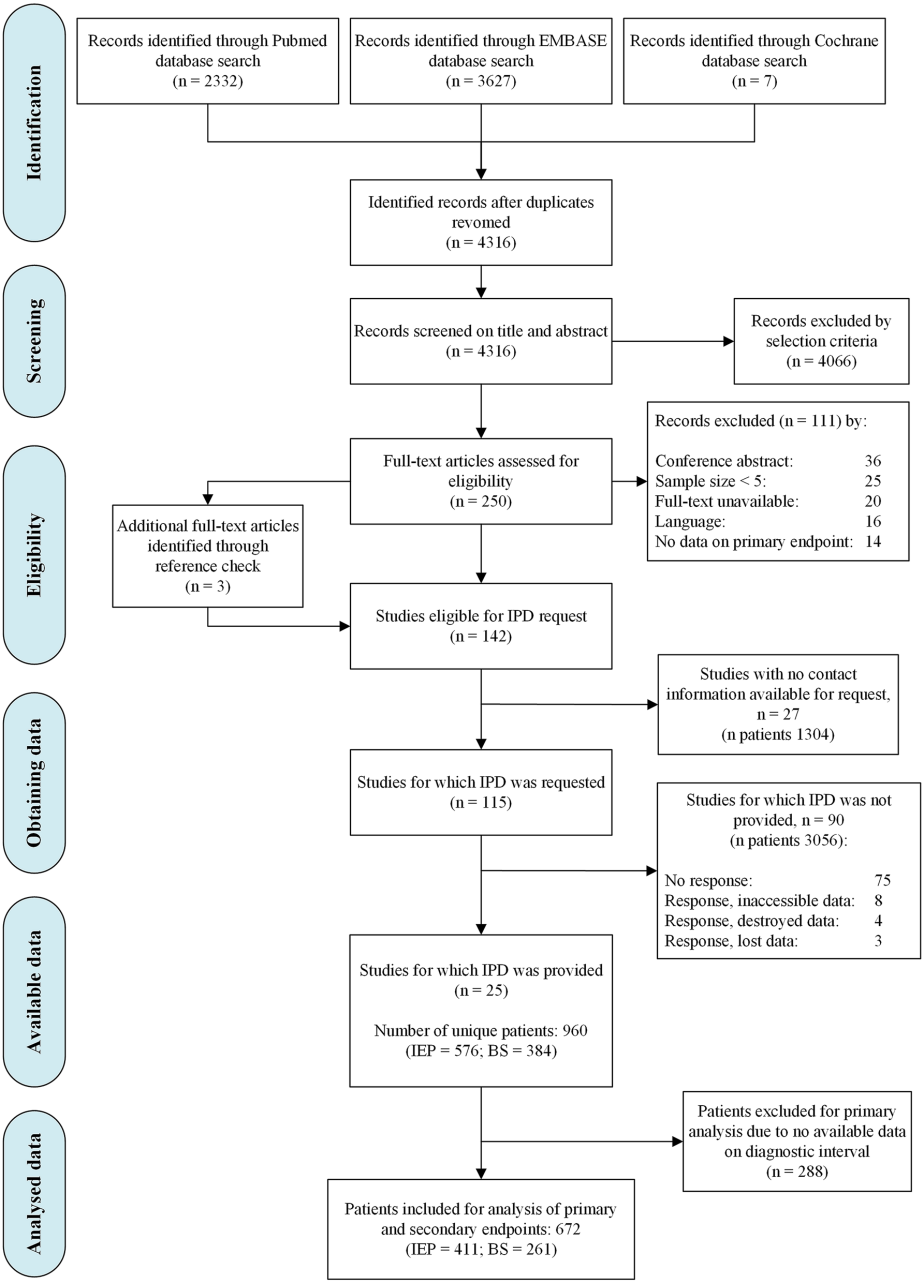
Flow chart of study and patient inclusion for the IPD meta-analysis. *IPD* individual patient data, *IEP* iatrogenic esophageal perforation, *BS* Boerhaave’s syndrome

The systematic search identified 2332, 3627 and 7 records from the Medline, Embase and Cochrane databases, respectively (Fig. 1). After removing duplicates, 4316 records remained, of which 4066 were excluded after screening the title and abstract. Subsequently, full-text assessment of the remaining 250 eligible articles resulted in 139 eligible studies. Reference cross-check of these studies yielded 3 additional eligible studies. Finally, a total of 142 studies were selected for inquiry of individual patient data (IPD).

### Individual patient data acquisition and quality assessment

We invited the corresponding and senior authors of selected studies per e-mail to share individual data of only patients with IEP and BS (Supplementary Table 5). All studies of which IPD was shared for meta-analysis were critically appraised with the Methodological Index for Non-Randomized Studies (MINORS) tool [16]. As supported by the tool designers, we only used the first 8 items for quality assessment because our data set comprised only non-comparative observational studies (Supplementary Table 4a). The items were scored as 0 (not reported), 1 (reported but inadequate) or 2 (reported and adequate).

### Study endpoints and definitions

We investigated whether TOD was associated with clinical outcomes in patients with IEP, and BS. TOD was defined as time between symptom onset and perforation diagnosis, measured in hours. We compared patients diagnosed ≤ 24 vs. > 24 h after onset, as well as patients diagnosed ≤ 12 vs. > 12 h after onset. We selected the 12- and 24-h thresholds to demonstrate the clinical importance of diagnosing an esophageal perforation at an early stage of the perforation. The primary endpoint of the study was overall mortality (i.e., all-cause mortality during follow-up). Secondary endpoints were length of hospital stay, need for re-interventions and intensive care unit (ICU) admissions for management of esophageal perforation. In addition, we assessed differences in therapeutic management and clinical outcomes between IEP and BS.

Additional endpoints were whether TOD was associated with clinical outcomes in patients treated with surgical or endoscopic interventions. Furthermore, we assessed risk factors for overall mortality during follow-up of patients with IEP and BS. Risk factors assessed included age (< 70 vs ≥ 70 years), gender, location of perforation and initial treatment (surgery, endoscopy, conservative or a combination of surgery and endoscopy).

Benign esophageal perforation was defined as a full-thickness rupture of the esophageal wall, caused by either (1) IEP following a therapeutic (or diagnostic) endoscopic procedure; or (2) BS (i.e., spontaneous esophageal rupture). Location of perforation was subdivided in the proximal/thoracic (i.e., proximal and middle one-third) and distal/abdominal (i.e., distal one-third) esophagus. Therapeutic approach was subdivided into surgical, endoscopic and conservative. Surgical approach was defined as any surgical intervention to treat the perforation (e.g., primary repair, video-assisted thoracic surgery with or without drain placement, esophagectomy). Endoscopic approach was defined as any endoscopic intervention to close the perforation (e.g., stent placement, clip placement, endoscopic vacuum therapy). Conservative approach was defined as primary treatment with ≥ 1 of the following supportive treatments for management of esophageal perforation: fasting (“nil by mouth”); enteral tube feeding; oral or intravenous antibiotics; percutaneous thoracic drain placement. All other study definitions are listed in the supplementary files (Supplementary Table 3).

### Statistical analysis

The model used for primary, secondary and additional endpoints used a meta-analytical effect estimate that was derived from the source data of all studies simultaneously. Patients with missing data on TOD were excluded from this analysis.

As advised by the PRISMA-IPD guidelines [15], we used a mixed effects model with random intercepts and slopes to account for clustering of patients within studies. We used a multivariable logistic regression model, introducing baseline parameters that significantly differed (p-value < 0.2) in the univariable analysis. Multivariable logistic regression was performed with backward stepwise elimination until all remaining variables reached a p-value of < 0.05. Results were expressed as percentages, odds ratio (OR) with 95% CI and significance levels and depicted in forest plots. To test whether TOD was associated with log-transformed length of hospital stay, we used a linear mixed effects model, introducing baseline parameters that significantly differed (p-value < 0.2) in the univariable analysis and performed backward stepwise elimination until all remaining variables reached a p-value of < 0.05. Differences in length of hospital stay were expressed as percentages and significance levels.

Multivariable Cox regression analysis was performed to calculate overall mortality hazard ratios (HR) with 95% confidence intervals (CI) while adjusting for the potential confounders age, gender, etiology, esophageal location of perforation and initial treatment modality. Difference in mortality between IEP and BS is shown with a Kaplan–Meier survival curve.

Differences in therapeutic management and clinical outcomes between patients with IEP and BS were assessed with Chi-square and t tests for categorical and continuous data, respectively. Survival was calculated from the date of perforation diagnosis to the date of death, plotted using Kaplan–Meier curves and compared using a log-rank test.

A two-tailed *p*-value of < 0.05 was considered significant in all statistical analyses. SPSS version 25.0 (IBM Corp, IBM SPSS Statistics for Windows, Armonk, NY) was used for study analyses.

## Results

### Individual patient data collection

The systematic review yielded 142 studies that met the study selection criteria for IPD acquisition. The characteristics of all eligible studies can be found in the supplementary files (Supplementary Table 5). For 27 studies no contact information was available. Therefore, authors of 115 studies were invited to share their IPD (Fig. 1).

In total, the IPD was based on 25 studies that were published between 2004 and 2017 (Supplementary Table 6). Authors of five cohorts included unpublished data of 58 patients and confirmed that the additional data were collected in accordance with the methodology of the original study [13, 17–20]. We found no inconsistencies between shared and published data. Supplementary Table 6 shows the study inclusion period and patient characteristics of all 25 included databases.

### Risk of bias assessment

We assessed the risk of bias in all 25 included studies (Supplementary Table 4b). The mean MINORS score was 6.8 (SD ± 1.7, range 3–10). All studies collected the data retrospectively (item #3), except for one study that was based on prospective data collection [21]. None of the studies reported on observer (item #5) or sample size bias (item #8).

### Baseline characteristics

In total, individual raw data of 960 patients with IEP (*n* = 576) and BS (*n* = 384) were included in the study. The baseline characteristics of all patients and differences between IEP and BS are shown in Table 1.

**Table 1:** Baseline characteristics and unadjusted differences between patients with IEP and BS

Variables	All patients	IEP	BS	*P* value
	(n = 960)	(n = 576)	(n = 384)	
Age, years (mean ± SD)	64 (18)	63 (18)	64 (17)	0.320
Gender: male, *n* (%)	617 (65)	333 (58)	284 (74)	**< 0.001**
Location perforation, *n* (%)				**< 0.001**
Proximal	362 (38)	296 (52)	66 (17)	
Distal	594 (62)	279 (49)	315 (83)	
Initial treatment, *n* (%)				
Surgery	501 (52)	269 (47)	232 (60)	**< 0.001**
Endoscopy	191 (20)	139 (24)	52 (14)	**< 0.001**
Conservative only	194 (20)	138 (24)	56 (15)	**< 0.001**
Surgery and endoscopy	73(8)	30 (5)	43 (11)	**0.001**
Outcome				
Overall mortality, *n* (%)	120 (13)	55 (10)	65 (17)	**0.001**
ICU admission, *n* (%)	531 (68)	264 (57)	267 (86)	**< 0.001**
Re-intervention, n (%)	289 (32)	143 (26)	146 (40)	**< 0.001**
LOS, median days (IQR)	18 [9–35]	15 [8–28]	27 [13–47]	**< 0.001**

Values represent number of patients (percentage of total in column) [n (%)], mean (SD), or median (IQR). Bold *p*-values indicate that differences between the groups were statistically significant

*n* number of patients, *SD* standard deviation, *IEP* iatrogenic esophageal perforation, *BS* Boerhaave’s syndrome, *ICU* intensive care unit, *LOS* length of hospital stay, *TOD* time of diagnosis, *IQR* interquartile range

In patients with IEP, the mean age was 63 years (SD ± 18), 333 (58%) were male and in 279 (49%) the perforation was located in the distal esophagus. After IEP diagnosis, initial therapeutic management consisted of surgery in 269 (47%) patients, endoscopy in 139 (24%), surgery and endoscopy in 30 (5%) and conservative treatment in 138 (24%). Following initial management, a total of 264 (57%) patients were admitted to the ICU and 143 (26%) required one or more re-interventions for perforation management. Median follow-up was 180 days (IQR 90–1521). A total of 55 (10%) patients died as a result of IEP. Median time to death was 37 days (IQR 13–90).

In patients with BS, the mean age was 64 years (SD ± 17), 284 (74%) were male and in 315 (83%) the perforation was located in the distal esophagus. After BS diagnosis, initial therapeutic management consisted of surgery in 232 (60%) patients, endoscopy in 52 (14%), surgery and endoscopy in 43 (11%) and conservative treatment in 56 (15%). Following initial management, a total of 267 (86%) patients were admitted to the ICU and 146 (40%) required one or more re-interventions for management of perforation. Median follow-up was 131 days (IQR 63–775). A total of 65 (17%) patients died as a result of BS. Median time to death was 20 days (IQR 6–46).

**Fig. 2:**
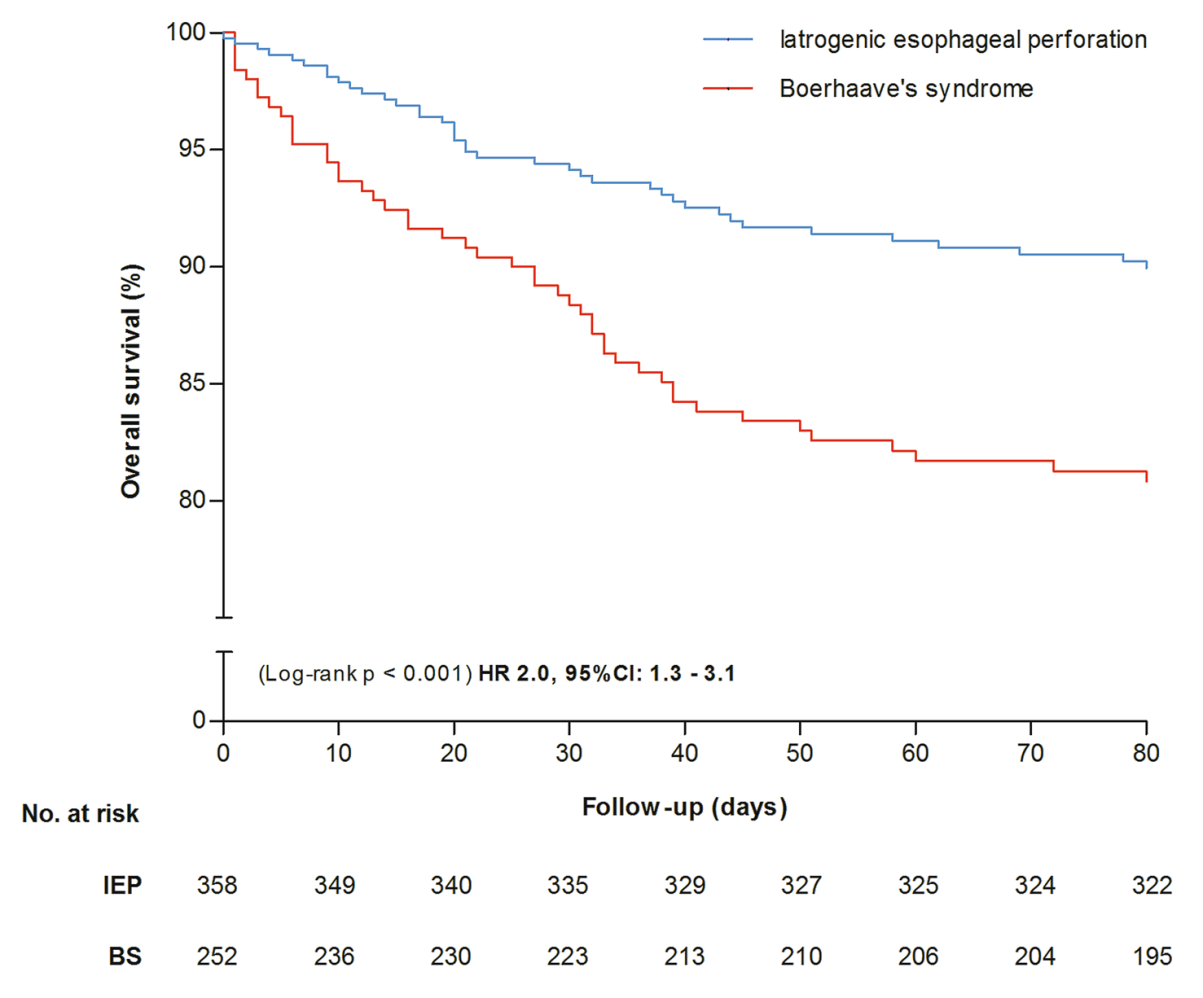
Survival differences between patients with IEP and BS during 3 months of follow-up. Hazard ratio is adjusted for age, gender, etiology, perforation location and initial treatment strategy. *IEP* iatrogenic esophageal perforation, *BS* Boerhaave’s syndrome

Figure 2 shows survival differences during the first 3 months after diagnosis between patients with IEP and BS. Cox regression analysis showed that, after adjustment for confounders, IEP was associated with decreased overall mortality when compared with BS (10% vs. 17%, HR 2.0, 95% CI 1.3–3.1). Data were missing on TOD in 288 (30%) patients. Therefore, we included 672/960 (70%) patients for analysis of the primary and secondary endpoints. Differences in age and initial treatment (surgery, endoscopy) between included and excluded patients were observed (Supplementary Table 7). Table 2 shows differences between baseline characteristics and outcome for early and late TOD (12 h and 24 h) in patients with IEP and BS.

**Table 2:** Unadjusted differences in baseline characteristics and clinical outcome between early and late TOD in patients with IEP and BS

	Iatrogenic esophageal perforation (n = 411)					*p*	Boerhaave’s syndrome (n = 261)					*p*
TOD:	≤ 12 h	> 12 h		≤ 24 h	> 24 h		≤ 12 h	> 12 h		≤ 24 h	> 24 h	
Characteristics	n = 257	n = 134	*p*	n = 323	n = 88		n = 101	n = 149	*p*	n = 149	n = 117	
Age, mean years (± SD)	61 (18)	59 (18)	0.197	63 (19)	58 (16)	**0.042**	64 (18)	59 (15)	0.022	66 (17)	58 (16)	**< 0.001**
Gender: male, *n* (%)	142 (55)	79 (59)	0.483	143 (44)	36 (41)	0.573	78 (77)	106 (71)	0.284	106 (74)	86 (74)	0.984
Location perforation, *n* (%)			0.232			0.057			**< 0.001**			**< 0.001**
Proximal	141 (55)	65 (49)	–	180 (56)	39 (44)	–	4 (4)	29 (20)	–	6 (4)	27 (23)	–
Distal	116 (45)	69 (51)	–	143 (44)	49 (56)	–	97 (96)	120 (81)	–	138 (96)	90 (77)	–
Initial treatment, n (%)												
Surgery	111 (43)	47 (35)	0.121	141 (44)	34 (39)	0.399	66 (65)	82 (55)	0.104	93 (65)	66 (56)	0.178
Endoscopy	74 (29)	38 (28)	0.928	90 (28)	25 (28)	0.920	12 (12)	23 (15)	0.427	16 (11)	19 (16)	0.227
Conservative only	64 (25)	35 (26)	0.793	76 (24)	23 (26)	0.612	9 (9)	29 (20)	**0.023**	14 (10)	24 (21)	**0.014**
Surgery and endoscopy	8 (3)	14 (10)	**0.003**	16 (5)	6 (7)	0.491	13 (13)	15 (10)	0.490	20 (14)	8 (7)	0.067

Bold *p*-values indicate that differences between the groups were statistically significant

*n* number of patients, *TOD* time of diagnosis, *h* hour, *SD* standard deviation

### Time of diagnosis and clinical outcome of IEP

**Fig. 3:**
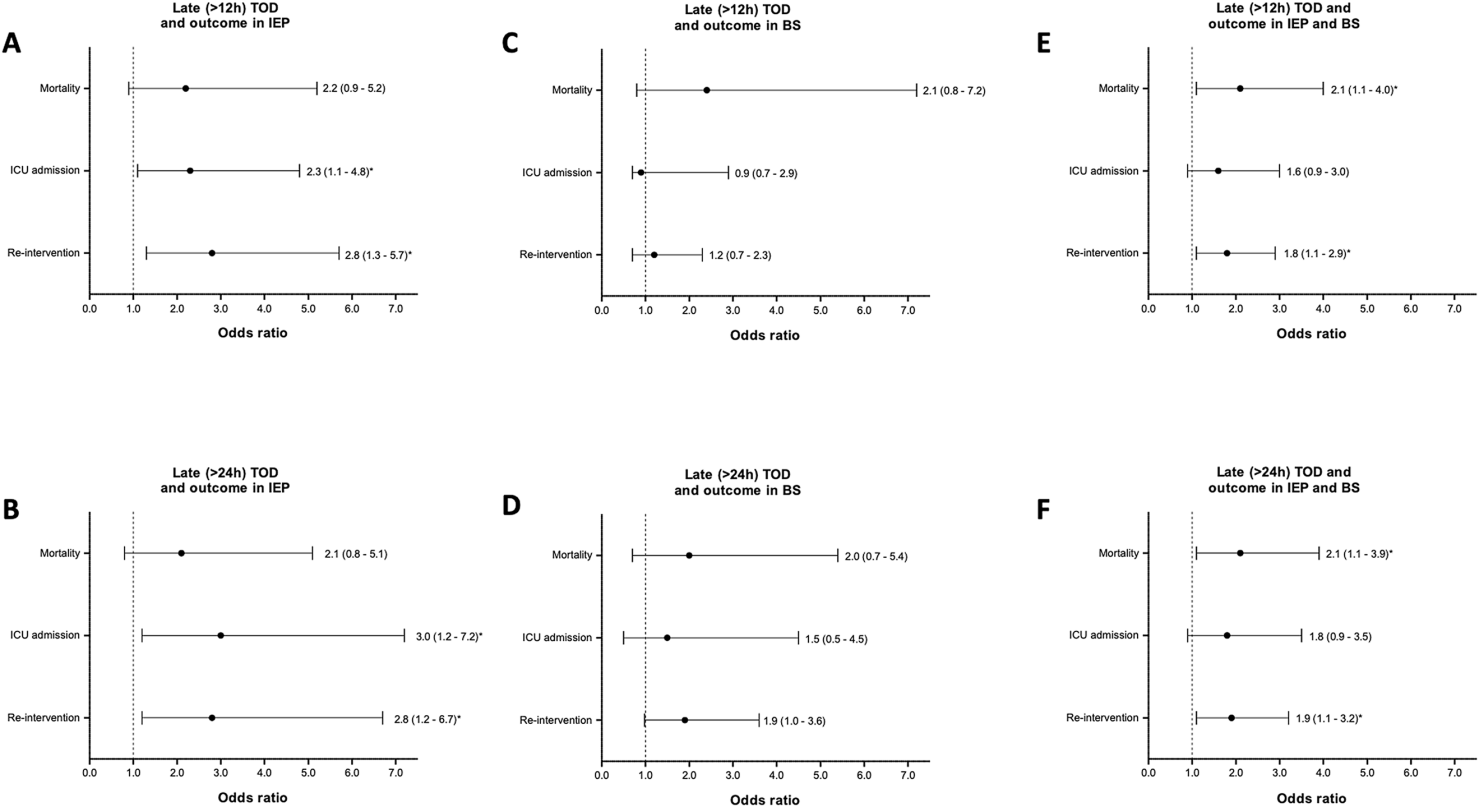
Forest plots depicting odds ratio of late TOD for overall mortality, ICU admission and re-intervention in patients with IEP and BS. Values represent odds ratios with 95% confidence intervals. Odds ratios are adjusted for treatment center, etiology, age, gender, perforation location and initial treatment approach. **A** Late (> 12 h) TOD and outcome in patients with IEP. **B** Late (> 24 h) TOD and outcome in patients with IEP. **C** Late (> 12 h) TOD and outcome in patients with BS. **D** Late (> 24 h) TOD and outcome in patients with BS. **E** Late (> 12 h) TOD and outcome in patients with IEP and BS. **F** Late (> 24 h) TOD and outcome in patients with IEP and BS. *TOD* time of diagnosis, *IEP* iatrogenic esophageal perforation, *BS* Boerhaave’s syndrome, *ICU* intensive care unit, *h* hour. *Indicates a statistically significant association

We assessed whether TOD was associated with clinical outcomes in patients with IEP (Table 3 and Fig. 3). Analysis of primary outcome in the 24 h subgroup showed that early TOD was not associated with overall mortality compared with late TOD (8% vs. 13%, OR 2.1, 95% CI 0.8–5.1). Analysis of the 12 h subgroup showed a similar trend, with no association between early TOD and overall mortality compared with late TOD (7% vs. 12%, OR 2.2, 95% CI 0.9–5.2).

**Table 3:** Multi-level multivariable logistic regression analysis for clinical outcome between early and late TOD in patients with IEP and BS

IEP (n = 411)	TOD (12 h)			Adjusted*			TOD (24 h)			Adjusted*		
	≤ 12 h n = 257	> 12 h n = 134	*p*	OR	95%CI	*p*	≤ 24 h n = 323	> 24 h n = 88	*p*	OR	95%CI	*p*
Outcome, n (%)												
Mortality	17 (7)	16 (12)	0.072	2.2	0.9–5.2	0.067	25 (8)	11 (13)	0.161	2.1	0.8–5.1	0.116
ICU admission	92 (44)	78 (65)	**< 0.001**	2.3	1.1–4.8	**0.033**	113 (46)	57 (69)	**< 0.001**	3.0	1.2–7.2	**0.017**
Re-intervention	49 (21)	56 (46)	**< 0.001**	2.8	1.3–5.7	**0.006**	69 (23)	38 (45)	**< 0.001**	2.8	1.2–6.7	**0.023**
LOS, days [IQR]	13 [6–21]	20 [12–34]	**< 0.001**	–	–	**< 0.001**	14 [7–23]	22 [12–34]	**< 0.001**	–	–	**< 0.001**

BS (n = 261)	TOD (12 h)		*p*	Adjusted*			TOD (24 h)		*p*	Adjusted*		
	≤ 12 h n = 101	> 12 h n = 149		OR	95%CI	*p*	≤ 24 h n = 144	> 24 h n = 117		OR	95%CI	*p*
Outcome, *n* (%)												
Mortality	12 (12)	27 (18)	0.182	2.4	0.8–7.2	0.104	21 (15)	21 (18)	0.462	2.0	0.7–5.4	0.167
ICU admission	70 (90)	117 (82)	0.144	0.9	0.7–2.9	0.887	95 (88)	92 (82)	0.227	1.5	0.5–4.5	0.475
Re-intervention	34 (37)	60 (42)	0.458	1.2	0.7–2.3	0.520	44 (33)	51 (46)	0.051	1.9	1.0–3.6	0.059
LOS, days [IQR]	24[12–41]	25 [11–45]	0.675	–	–	0.721	25[12–43]	23 [11–44]	0.745	–	–	0.710

IEP + BS (n = 672)	TOD (12 h)			Adjusted^†^			TOD (24 h)			Adjusted^†^		
	≤ 12 h n = 358	> 12 h n = 283	*p*	OR	95%CI	*p*	≤ 24 h n = 467	> 24 h n = 205	*p*	OR	95%CI	*p*
Outcome, n (%)												
Mortality	29 (8)	43 (15)	**0.005**	2.1	1.1–4.0	**0.019**	46 (10)	32 (16)	**0.032**	2.1	1.1–3.9	**0.026**
ICU admission	162 (57)	195 (74)	**< 0.001**	1.6	0.9–3.0	0.114	208 (59)	149 (76)	**< 0.001**	1.8	0.9–3.5	0.084
Re-intervention	83 (25)	116 (44)	**< 0.001**	1.8	1.1–2.9	**0.022**	113 (26)	89 (45)	**< 0.001**	1.9	1.1–3.2	**0.026**
LOS, days [IQR]	14 [7–26]	22 [12–39]	**< 0.001**	–	–	** < 0.001**	16 [8–30]	22 [12–38]	**< 0.001**	–	–	**0.001**

Bold *p*-values indicate that differences between the groups were statistically significant

*Analysis is adjusted for age, gender, location of perforation, initial treatment strategy and center of treatment

^†^Analysis is adjusted for type of perforation (etiology), age, gender, location of perforation, initial treatment strategy and center of treatment

Numbers of patients within 12 h interval do not always add up to the total number of patients owing to missing diagnostic interval data in 31 (5%) patients with IEP and BS

*n* number of patients, *TOD* time of diagnosis, *IEP* iatrogenic esophageal perforation, *BS* Boerhaave’s syndrome, *h* hour, *OR* odds ratio, *CI* confidence interval, *ICU* intensive care unit, *LOS* length of stay (median), *IQR* interquartile range

Analysis of secondary outcomes in the 24 h subgroup showed that early TOD was associated with a 23% decrease in ICU admissions (46% vs. 69%, OR 3.0, 95% CI 1.2–7.2), a 22% decrease in need for re-interventions (23% vs. 45%, OR 2.8, 95% CI 1.2–6.7) and a 36% decrease in mean length of hospital stay 14 vs. 22 days, *p* < 0.001), compared with late TOD. Analysis of the 12 h subgroup showed that early TOD was associated with a 21% decrease in ICU admissions (44% vs. 65%, OR 2.3, 95% CI 1.1–4.8), a 25% decrease in need for re-interventions (21% vs. 46%, OR 2.8, 95% CI 1.3–5.7) and a 35% decrease in mean length of hospital stay (13 vs. 20 days, *p* < 0.001), compared with late TOD.

### Time of diagnosis and clinical outcome of BS

We also assessed whether TOD was associated with clinical outcomes in patients with BS (Table 3 and Fig. 3). Analysis of the 24 h subgroup showed that early TOD was not associated with overall mortality compared with late TOD (15% vs. 18%, OR 2.0, 95% CI 0.7–5.4). Analysis of the 12 h subgroup showed that early TOD was also not associated with overall mortality compared with late TOD (12% vs. 18%, OR 2.4, 95% CI 0.8–7.2).

Analysis of secondary outcomes in the 24 h subgroup showed that early TOD was not associated with fewer ICU admissions (88% vs. 82%, OR 1.5, 95% CI 0.5–4.5), fewer re-interventions (33% vs. 46%, OR 1.9, 95% CI 0.98–3.6) or shorter length of hospital stay (25 vs. 23 days, *p* = 0.710), compared with late TOD. Analysis of the 12 h subgroup similarly showed that early TOD was not associated with fewer ICU admissions (90% vs. 82%, OR 0.9, 95% CI 0.7–2.9), fewer re-interventions (37% vs. 42%, OR 1.2, 95% CI 0.7–2.3) or shorter length of hospital stay (24 vs. 25 days, *p* = 0.721), compared with late TOD.

### Time of diagnosis and clinical outcome of IEP and BS

We assessed whether TOD was associated with clinical outcomes when combining results of IEP and BS in a multivariable analysis, with adjustment for type of perforation and other confounders (Table 3 and Fig. 3). Analysis of the 24 h subgroup showed that early TOD was associated with a 6% decrease in overall mortality compared with late TOD (10% vs. 16%, OR 2.1, 95% CI 1.1–3.9). Analysis of the 12 h subgroup showed that early TOD was similarly associated with a 7% decrease in overall mortality compared with late TOD (8% vs. 15%, OR 2.1, 95% CI 1.1–4.0).

Analysis of the secondary outcomes in the 24 h subgroup showed that early TOD was associated with a 35% decrease in mean length of hospital stay (16 vs. 22 days, *p* = 0.001), a 19% decrease in need for re-interventions (26% vs. 45%, OR 1.9, 95% CI 1.1–3.2) and no difference in ICU admission (59% vs. 76%, OR 1.8, 95% CI 0.9–3.5) compared with late TOD. Analysis of the 12 h subgroup showed that early TOD was associated with a 28% decrease in mean length of hospital stay (14 vs. 22 days, *p* < 0.001), a 19% decrease in need for re-intervention (25% vs. 44%, OR 1.8, 95% CI 1.1–2.9) and no difference in ICU admissions (57% vs. 74%, OR 1.6, 95% CI 0.9–3.0) compared with late TOD.

### Additional endpoints

We assessed whether TOD was associated with clinical outcomes in patients initially treated with surgical or endoscopic interventions. Results are shown in Table 4. In summary, we found no association between TOD and overall mortality in patients initially treated with a surgical or an endoscopic intervention.

**Table 4:** Multi-level multivariable regression analysis comparing early vs. late time of diagnosis of benign esophageal perforation in patients treated with endoscopic intervention only or surgical interventions only

Endoscopy	TOD (12 h)			Adjusted*			TOD (24 h)			Adjusted*		
(n = 150)	≤ 12 h n = 86	> 12 h n = 61	*p*	OR	95%CI	*p*	≤ 24 h n = 106	> 24 h n = 44	*p*	OR	95%CI	*p*
Outcome, n (%)												
Mortality	7 (8)	10 (16)	0.123	2.2	0.6–7.5	0.217	9 (9)	8 (18)	0.088	2.8	0.8–10.2	0.118
ICU admission	30 (39)	42 (74)	** < 0.001**	6.8	2.3–19.7	**0.001**	40 (43)	32 (76)	** < 0.001**	4.6	1.5–13.7	**0.007**
Re-intervention	26 (32)	35 (60)	**0.001**	4.6	1.8–11.8	**0.002**	33 (33)	29 (67)	** < 0.001**	4.7	1.8–12.3	**0.002**
LOS, days [IQR]	11 [6–22]	26 [16–41]	** < 0.001**	–	–	** < 0.001**	13 [7–22]	29 [17–42]	** < 0.001**	–	–	** < 0.001**

Surgery	TOD (12 h)			Adjusted*			TOD (24 h)			Adjusted*		
(n = 324)	≤ 12 h n = 177	> 12 h n = 129	*p*	OR	95%CI	*p*	≤ 24 h n = 234	> 24 h n = 100	*p*	OR	95%CI	*p*
Outcome, n (%)												
Mortality	13 (7)	16 (12)	0.136	2.1	0.9–4.7	0.072	24 (10)	11 (11)	0.839	1.4	0.6–3.2	0.396
ICU admission	104 (87)	101 (88)	0.920	0.8	0.3–2.2	0.667	51 (25)	41 (44)	**0.001**	0.8	0.3–2.3	0.637
Re-intervention	38 (24)	52 (44)	** < 0.001**	2.2	1.2–4.1	**0.011**	125 (88)	80 (87)	0.808	1.8	0.9–3.6	0.072
LOS, days [IQR]	17 [12–30]	25 [14–46]	**0.006**	–	–	**0.001**	19[12–35]	13 [13–44]	0.247	–	–	0.135

Bold *p*-values indicate that differences between the groups were statistically significant

Numbers of patients within 12 h interval do not always add up to the total number of patients owing to missing diagnostic interval data in 31 (5%) patients with IEP and BS

*n* number of patients, *TOD* time of diagnosis, *IEP* iatrogenic esophageal perforation, *BS* Boerhaave’s syndrome, *h* hour, *OR* odds ratio, *CI* confidence interval, *ICU* intensive care unit, *LOS* length of stay in the hospital (median), *IQR* interquartile range

*Analysis is adjusted for age, gender, location of perforation, etiology of benign esophageal perforation and center of treatment

Furthermore, we assessed risk factors associated with overall mortality in patients with IEP or BS. Results are shown in Table 5. Multivariable analysis identified age ≥ 70 to be associated with overall higher mortality in patients with IEP and BS. In addition, surgery, endoscopic and conservative treatment were all significantly associated with overall higher mortality in patients with IEP.

**Table 5:** Multi-level multivariable logistic regression analysis of risk factors for overall mortality during follow-up of patients with IEP or BS

	IEP (n = 576)			Adjusted*			BS (n = 384)			Adjusted*		
Risk factor	Yes	No	*p*	OR	95%CI	*p*	Yes	No	*p*	OR	95%CI	*p*
Overall mortality												
Age ≥ 70	35 (15%)	20 (6%)	**< 0.001**	2.9	1.5–5.7	**0.002**	41 (30%)	24 (10%)	**< 0.001**	5.9	3.0–11.5	**< 0.001**
Male gender	35 (11%)	20 (8%)	0.358	1.5	0.8–2.7	0.197	52 (18%)	13 (13%)	0.223	0.5	0.3–1.1	0.107
Proximal location	23 (8%)	32 (12%)	0.132	1.8	1.0–3.3	0.064	13 (20%)	51 (16%)	0.488	1.0	0.4–2.5	0.975
Initial treatment												
Surgery only	28 (10%)	27 (9%)	0.511	0.3	0.1–0.9	**0.026**	32 (14%)	33 (22%)	**0.043**	1.2	0.4–3.7	0.811
Endoscopy only	10 (7%)	45 (10%)	0.278	0.2	0.1–0.7	**0.011**	8 (15%)	57 (17%)	0.750	0.9	0.2–3.5	0.913
Conservative only	10 (7%)	45 (10%)	0.291	0.3	0.1–0.9	**0.028**	20 (36%)	45 (14%)	**< 0.001**	3.4	0.9–12.3	0.060
Surgery and endoscopy	7 (23%)	48 (9%)	**0.008**	–	–	–	5 (12%)	60 (18%)	0.325	–	–	–

Bold *p*-values indicate that differences between the groups were statistically significant

−Could not be estimated due to limited data

*n*, number of patients, *IEP* iatrogenic esophageal perforation, *BS* Boerhaave’s syndrome, *OR* odds ratio, *CI* confidence interval, *ICU* intensive care unit

*Analysis is adjusted for age, gender, location of perforation, initial treatment modality and center of treatment

## Discussion

This IPDMA included 960 patients with esophageal perforation from 25 retrospective cohorts. Whereas in patients with IEP or BS, the association between TOD and mortality was found to be not statistically significant, combining the results of IEP and BS showed a reduced overall mortality within 12 to 24 h after diagnosis. In addition, either separately or combined, early diagnosis of IEP and BS was associated with a reduction in ICU admissions, re-interventions and length of hospital stay.

Overall mortality in our IPDMA was 13% which corresponds with the pooled mortality (11%) from the included but not having responded 117 eligible studies in the current systematic review (Supplementary Table 5). In line with this, this percentage reflects the pooled mortality (12%) reported in a conventional meta-analysis of 75 studies [7].

Our study provides an interesting insight into the existing literature on esophageal perforation. Previous studies predominantly consisted of uncontrolled, retrospective evaluations of esophageal perforation, irrespective of type of perforation. Approximately three-quarters of the 142 studies yielded by our systematic literature review (Supplementary Table 5), also included patients with malignant, external traumatic or intra-operative perforations. The fact that IEP, BS and the remaining types of perforation vary significantly makes the interpretation of study findings and translation to daily clinical practice difficult. As a result, clinicians largely rely on their own clinical experience and on the expert opinion-based literature on management of patients with esophageal perforation [5, 12, 13, 22, 23].

Risk factors for mortality in patients with esophageal perforation have been previously assessed and include age, co-morbidity (mainly cardiovascular, liver and renal disease), etiology (i.e., BS) and initial treatment with endoscopic interventions [6]. Some studies have suggested that TOD is also a risk factor for adverse clinical outcome, but so far no studies clearly have demonstrated an independent association in patients with esophageal perforation [5, 9–13, 24]. This may be explained by at least two factors. First, as mentioned above, studies generally consist of small case series (range 27–119 patients [5, 9–13, 24]) from one center, which allows authors to perform only univariable analyses without accounting for type of perforation and treatment strategy. Second, treatment options selected for the management of esophageal perforation in these studies vary considerably within and between studies, which also causes heterogeneity (Supplementary Table 5) [25, 26]. In an effort to overcome these shortcomings, we pooled individual data from almost a thousand patients and performed multivariable regression analysis. This allowed to stratify clinical outcome by type of perforation and also to adjust for confounding.

In the present study, we hypothesized that early TOD (within 12–24 h) improves overall survival in patients with IEP and BS. To test this hypothesis, we adjusted for several factors (e.g., center of treatment, type of initial management) that could influence clinical outcome. Despite this, we only found a trend favoring early diagnosis when studying the association between TOD and overall mortality in individual causes of benign esophageal perforation (IEP: *p* = 0.067 and BS: *p* = 0.104). The absence of this association is likely explained by a limited statistical power, but the combined results of IEP and BS showed a much stronger benefit with regard to overall outcome measures. Nonetheless, this pooled analysis should be interpreted with caution as the results cannot be directly translated to clinical practice as we also showed that IEP and BS are clinically different conditions. Furthermore, it should be kept in mind, that for a complex multifactorial clinical problem like esophageal perforation, other possible prognostic factors, for example admission or transfer to a high volume center with experience in multidisciplinary care may adversely impact timely management but also reduce the mortality risk [27]. When establishing TOD as an independent risk factor for mortality in a multivariable analysis, adjustment for center of treatment or other factors will likely still confound the outcome.

Nevertheless, the question remains how a diagnosis of esophageal perforation can be accelerated. In patients with IEP, careful monitoring for signs of esophageal perforation may be helpful as it particularly may occur in therapeutic upper endoscopic gastrointestinal procedures [1]. Although monitoring of patients for adverse events after therapeutic interventions of the upper gastrointestinal tract is advised by the European Society for Gastrointestinal Endoscopy (ESGE) [28], IEP is still frequently missed during the first 24 h after endoscopy. This is supported by our observation that IEP was diagnosed after 12 h in one-third (33%) and after 24 h in one-fifth (21%) of patients. Close observation for at least 12 h after therapeutic esophageal procedures is therefore strongly recommended [28–30]. In contrast to IEP, early diagnosis of BS likely will remain a major challenge in clinical practice as it usually occurs in an out-of-hospital setting and often mimics various other acute conditions (e.g., ischemic cardiac disease) [31].

The main strength of this study is that we were able to use original source data. First, participating authors reviewed and shared their original data according to our pre-specified study definitions. We were able to set up a database with uniformly defined parameters without being restricted by divergent definitions used in the original studies. Furthermore, this systematic approach allowed obtaining additional unpublished data that were required for analysis of study endpoints.

Second, the data set allowed performing a multivariable analysis while correcting for the established confounders age and therapeutic approach. We further included potential confounders including gender, esophageal location, initial conservative treatment and type of treatment center. Accounting for therapeutic approach seems also important. For example, patients treated conservatively either may have minor esophageal injury or, conversely, were in a poor medical condition and unfit for surgical or endoscopic interventions, likely resulting in death shortly after presentation [32].

There are also some limitations that should be recognized when interpreting the findings of this study. First, data on TOD were missing in a considerable number of patients (30%). Second, after critical appraisal, we estimated that the included studies had a high risk of bias due to their observational design. The relative rarity and various clinical presentations of esophageal perforation in daily clinical practice likely explains the lack of published prospective and randomized controlled trials. Moreover, randomizing patients in the acute setting is challenging [33]. Even by adjusting for important confounders, limitations in our retrospective data set did not allow to adjust for disease severity and co-morbidity of patients. We therefore recommend initiating nation-wide prospective registries that could generate high-level evidence on clinical management of esophageal perforation, preferably stratified by type of perforation. In addition, future research should focus on the multidimensional aspect of management of esophageal perforation rather than individual factors such as a particular treatment modality.

In conclusion, this IPD meta-analysis suggests that early diagnosis within 12 to 24 h after onset was associated with improved clinical outcome compared with a late diagnosis in patients with IEP and BS. Our findings confirm current opinion that these types of esophageal perforation are clinical emergencies that should be recognized as soon as possible.

## Electronic supplementary material

Below is the link to the electronic supplementary material.

Supplementary file1 (DOCX 247 kb)

## Abbreviations

TOD: Time of diagnosis
IEP: Iatrogenic esophageal perforation
BS: Boerhaave’s syndrome
IPDMA: Individual patient data meta-analysis
LOS: Length of hospital stay
ICU: Intensive care unit
CI: Confidence interval
OR: Odds ratio
SD: Standard deviation
