# Supplementary material for: Early diagnosis is associated with improved clinical outcomes in benign esophageal perforation: an individual patient data meta-analysis
Source: Surg Endosc. 2020 Jul 17;35(7):3492–505. doi: 10.1007/s00464-020-07806-y (PMC8195755; doi:10.1007/s00464-020-07806-y)
Supplement: Supplementary file 1 — Supplementary file1 (DOCX 247 kb) [file 464_2020_7806_MOESM1_ESM.docx]

# SUPPLEMENTARY MATERIAL

# Appendix 1: PRISMA-IPD checklist.

| **Supplementary table 1: PRISMA-IPD checklist** | | |  |
| --- | --- | --- | --- |
| **PRISMA-IPD**  **Section/topic** | **Item No** | **Checklist item** | **Reported on page** |
| Title | | | |
| Title | 1 | Identify the report as a systematic review and meta-analysis of individual participant data. | 1 |
| Abstract | | | |
| Structured summary | 2 | Provide a structured summary including as applicable: | 2 |
|  |  | Background: state research question and main objectives, with information on participants, interventions, comparators and outcomes. |  |
|  |  | Methods: report eligibility criteria; data sources including dates of last bibliographic search or elicitation, noting that IPD were sought; methods of assessing risk of bias. |  |
|  |  | Results: provide number and type of studies and participants identified and number (%) obtained; summary effect estimates for main outcomes (benefits and harms) with confidence intervals and measures of statistical heterogeneity. Describe the direction and size of summary effects in terms meaningful to those who would put findings into practice. |  |
|  |  | Discussion: state main strengths and limitations of the evidence, general interpretation of the results and any important implications. |  |
|  |  | Other: report primary funding source, registration number and registry name for the systematic review and IPD meta-analysis. |  |
| Introduction | | | |
| Rationale | 3 | Describe the rationale for the review in the context of what is already known. | 4 |
| Objectives | 4 | Provide an explicit statement of the questions being addressed with reference, as applicable, to participants, interventions, comparisons, outcomes and study design (PICOS). Include any hypotheses that relate to particular types of participant-level subgroups. | 4, 5 |
| Methods | | | |
| Protocol and registration | 5 | Indicate if a protocol exists and where it can be accessed. If available, provide registration information including registration number and registry name. Provide publication details, if applicable. | 5 |
| Eligibility criteria | 6 | Specify inclusion and exclusion criteria including those relating to participants, interventions, comparisons, outcomes, study design and characteristics (e.g. years when conducted, required minimum follow-up). Note whether these were applied at the study or individual level i.e. whether eligible participants were included (and ineligible participants excluded) from a study that included a wider population than specified by the review inclusion criteria. The rationale for criteria should be stated. | 5 |
| Identifying studies - information sources | 7 | Describe all methods of identifying published and unpublished studies including, as applicable: which bibliographic databases were searched with dates of coverage; details of any hand searching including of conference proceedings; use of study registers and agency or company databases; contact with the original research team and experts in the field; open adverts and surveys. Give the date of last search or elicitation. | 5 |
| Identifying studies - search | 8 | Present the full electronic search strategy for at least one database, including any limits used, such that it could be repeated. | 5, Supplementary table 2 |
| Study selection processes | 9 | State the process for determining which studies were eligible for inclusion. | 6, Figure 1 |
| Data collection processes | 10 | Describe how IPD were requested, collected and managed, including any processes for querying and confirming data with investigators. If IPD were not sought from any eligible study, the reason for this should be stated (for each such study). | 6 |
|  |  | If applicable, describe how any studies for which IPD were not available were dealt with. This should include whether, how and what aggregate data were sought or extracted from study reports and publications (such as extracting data independently in duplicate) and any processes for obtaining and confirming these data with investigators. |  |
| Data items | 11 | Describe how the information and variables to be collected were chosen. List and define all study level and participant level data that were sought, including baseline and follow-up information. If applicable, describe methods of standardising or translating variables within the IPD datasets to ensure common scales or measurements across studies. | 6, 7,  Supplementary table 3 |
| IPD integrity | A1 | Describe what aspects of IPD were subject to data checking (such as sequence generation, data consistency and completeness, baseline imbalance) and how this was done. | 6 |
| Risk of bias assessment in individual studies. | 12 | Describe methods used to assess risk of bias in the individual studies and whether this was applied separately for each outcome. If applicable, describe how findings of IPD checking were used to inform the assessment. Report if and how risk of bias assessment was used in any data synthesis. | 6, 7,  Supplementary tables 4a+b |
| Specification of outcomes and effect measures | 13 | State all treatment comparisons of interests. State all outcomes addressed and define them in detail. State whether they were pre-specified for the review and, if applicable, whether they were primary/main or secondary/additional outcomes. Give the principal measures of effect (such as risk ratio, hazard ratio, difference in means) used for each outcome. | 6, 7, 8 |
| Synthesis methods | 14 | Describe the meta-analysis methods used to synthesise IPD. Specify any statistical methods and models used. Issues should include (but are not restricted to):  Use of a one-stage or two-stage approach.  How effect estimates were generated separately within each study and combined across studies (where applicable).  Specification of one-stage models (where applicable) including how clustering of patients within studies was accounted for.  Use of fixed or random effects models and any other model assumptions, such as proportional hazards.  How (summary) survival curves were generated (where applicable).  Methods for quantifying statistical heterogeneity (such as I^2^ and τ^2^).  How studies providing IPD and not providing IPD were analysed together (where applicable).  How missing data within the IPD were dealt with (where applicable). | 8, 9 |
| Exploration of variation in effects | A2 | If applicable, describe any methods used to explore variation in effects by study or participant level characteristics (such as estimation of interactions between effect and covariates). State all participant-level characteristics that were analysed as potential effect modifiers, and whether these were pre-specified. | 8 |
| Risk of bias across studies | 15 | Specify any assessment of risk of bias relating to the accumulated body of evidence, including any pertaining to not obtaining IPD for particular studies, outcomes or other variables. | n.a. |
| Additional analyses | 16 | Describe methods of any additional analyses, including sensitivity analyses. State which of these were pre-specified. | 7 |
| Results | | | |
| Study selection and IPD obtained | 17 | Give numbers of studies screened, assessed for eligibility, and included in the systematic review with reasons for exclusions at each stage. Indicate the number of studies and participants for which IPD were sought and for which IPD were obtained. For those studies where IPD were not available, give the numbers of studies and participants for which aggregate data were available. Report reasons for non-availability of IPD. Include a flow diagram. | 6, 9, Figure 1 |
| Study characteristics | 18 | For each study, present information on key study and participant characteristics (such as description of interventions, numbers of participants, demographic data, unavailability of outcomes, funding source, and if applicable duration of follow-up). Provide (main) citations for each study. Where applicable, also report similar study characteristics for any studies not providing IPD. | Supplementary table 6 |
| IPD integrity | A3 | Report any important issues identified in checking IPD or state that there were none. | 9 |
| Risk of bias within studies | 19 | Present data on risk of bias assessments. If applicable, describe whether data checking led to the up-weighting or down-weighting of these assessments. Consider how any potential bias impacts on the robustness of meta-analysis conclusions. | 9, 10, Supplementary tables 4a+b |
| Results of individual studies | 20 | For each comparison and for each main outcome (benefit or harm), for each individual study report the number of eligible participants for which data were obtained and show simple summary data for each intervention group (including, where applicable, the number of events), effect estimates and confidence intervals. These may be tabulated or included on a forest plot. | Supplementary table 6 |
| Results of syntheses | 21 | Present summary effects for each meta-analysis undertaken, including confidence intervals and measures of statistical heterogeneity. State whether the analysis was pre-specified, and report the numbers of studies and participants and, where applicable, the number of events on which it is based. | 11, 12,  Figures 2-4 |
|  |  | When exploring variation in effects due to patient or study characteristics, present summary interaction estimates for each characteristic examined, including confidence intervals and measures of statistical heterogeneity. State whether the analysis was pre-specified. State whether any interaction is consistent across trials. |  |
|  |  | Provide a description of the direction and size of effect in terms meaningful to those who would put findings into practice. |  |
| Risk of bias across studies | 22 | Present results of any assessment of risk of bias relating to the accumulated body of evidence, including any pertaining to the availability and representativeness of available studies, outcomes or other variables. | n.a. |
| Additional analyses | 23 | Give results of any additional analyses (e.g. sensitivity analyses). If applicable, this should also include any analyses that incorporate aggregate data for studies that do not have IPD. If applicable, summarize the main meta-analysis results following the inclusion or exclusion of studies for which IPD were not available. | Supplementary table 5 |
| Discussion | | | |
| Summary of evidence | 24 | Summarize the main findings, including the strength of evidence for each main outcome. | 12 |
| Strengths and limitations | 25 | Discuss any important strengths and limitations of the evidence including the benefits of access to IPD and any limitations arising from IPD that were not available. | 15, 16 |
| Conclusions | 26 | Provide a general interpretation of the findings in the context of other evidence. | 12, 13 |
| Implications | A4 | Consider relevance to key groups (such as policy makers, service providers and service users). Consider implications for future research. | 14 |
| Funding | | | |
| Funding | 27 | Describe sources of funding and other support (such as supply of IPD), and the role in the systematic review of those providing such support. | 16 |

**Appendix 2.** Search strategy for Pubmed, EMBASE and CENTRAL databases.

| **Supplementary table 2. Search strategy for Pubmed, EMBASE and Cochrane databases** | |
| --- | --- |
| **Terms** | **Synonyms** |
| “Esophageal perforation” | Boerhaave's syndrome OR Boerhave syndrome OR Boerhaave syndrome OR spontaneous perforation* OR spontaneous rupture* OR ((oesophag* OR esophag*) AND (perforation* OR rupture* OR tear* OR rent* OR leak*)) OR (((iatrogen* OR instrumental OR endoscopic) AND perforation* OR rupture*)) AND esophag*))) OR “Esophageal Perforation”[Mesh] OR “Boerhaave syndrome”[Mesh] |
| **AND** | |
| “Treatment” | antibiotic* OR non-operative OR nonoperative OR nutritional support OR diversion OR drainage OR non-surgical OR nonsurgical OR (conservative AND (treatment* OR therap* OR management*)) OR ((esophageal repair OR (surg* AND (treatment* OR therap* OR management* OR procedure*)) OR (operati* AND (treatment* OR therap* OR management*)) OR ((stent* OR ((esophagoscop* OR endoscop*) AND (procedure* OR management OR therap* OR treatment)) OR “Esophagus / surgery”[Mesh] OR “Esophageal Diseases / surgery”[Mesh] OR “Esophagoscopy”[Mesh] OR “Stents*”[Mesh] |
| **AND** | |
| “Clinical study” | randomized OR randomised OR RCT OR clinical trial* OR Cohort OR Concurrent Studies OR Concurrent Study OR Incidence Studies OR Incidence OR Follow-Up OR Longitudinal OR Prospective OR Retrospective OR Chart review* OR Chart stud* OR group OR case series OR "Cohort Studies"[Mesh] |

**Appendix 3.** Study definitions.

| **Supplementary table 3: Study definitions** | |
| --- | --- |
| Boerhaave’s syndrome | Spontaneous esophageal rupture |
| Iatrogenic esophageal perforation | Perforation after esophageal introduction of any endoscopic device or procedure |
| Malignant esophageal perforation | Perforation of a malignant esophageal lesion |
| External traumatic esophageal perforation | Perforation caused by external trauma (e.g. car accident, gunshot, stab wound) |
| Intra-operative esophageal perforation | Esophageal injury caused by intra-abdominal or thoracic surgery |
| Proximal esophagus | The proximal and middle part of the esophagus, also known as thoracic esophagus |
| Distal esophagus | The distal one-third of the esophagus |
| Conservative treatment | Patient is treated with ≥1 of the following supportive treatments for management of esophageal perforation:   - Fasting (“nil by mouth”); - Placement of enteral feeding tube, with or without endoscopic assistance; - Oral or intravenous antibiotics; - Percutaneous thoracic drainage, without VATS. |
| Endoscopic intervention | Any endoscopic intervention to close the esophageal perforation.  For example:   - Stent placement; - Clip placement; - Endoscopic vacuum therapy. |
| Surgical intervention | Any surgical intervention for management of esophageal perforation.  For example:   - Primary repair; - VATS with or without drain placement; - Esophagectomy. |
| Timing of diagnosis | The time between the onset of symptoms related to esophageal perforation - and the moment of diagnosis in the hospital. Time is measured in hours. |
| Primary outcome (mortality) | Esophageal perforation related death during follow-up. |
| Secondary outcomes | - ICU admission for management of esophageal perforation; - Re-intervention(s) for management of esophageal perforation; - LOS: length of hospital stay for management of esophageal perforation. Length is measured in days. |
| Re-intervention | Any of the following interventions performed after the initial intervention for management of esophageal perforation:   - Thoracic drainage of cavities; - Endoscopic drainage of cavities; - Esophageal stent placement; - Endoscopic clipping of the perforation; - Any surgical intervention. |
| IEP, iatrogenic esophageal perforation; BS, Boerhaave’s syndrome; VATS, video-assisted thoracic surgery; ICU, intensive care unit; LOS, length of hospital stay. | |

**Appendix 4.** MINORS assessment criteria for non-comparative studies.

| **Supplementary table 4a: MINORS assessment criteria for non-comparative studies** | | | | |
| --- | --- | --- | --- | --- |
|  | **Score** | **2** | **1** | **0** |
| **Methodological items** |  |  |  |  |
| **1. A clearly stated aim** |  | Aim or hypothesis including outcomes have been reported | Aim or hypothesis have been reported without a clear outcome | Not reported |
| **2. Inclusion of consecutive patients** |  | Explicit inclusion and exclusion criteria have been reported | Unclear or poor description inclusion and exclusion criteria have been reported | Not reported |
| **3. Prospective collection of data** |  | Prospective | Retrospective | Not reported |
| **4. Endpoints appropriated to the aim of the study** |  | Outcomes are appropriate to the aim of the study | Outcomes are not appropriate to the aim of the study | Not reported |
| **5. Unbiased assessment of the study endpoint** |  | Blind evaluation of objective outcomes and double-blind evaluation of subjective outcomes | One or more outcomes have been blinded | Blinding has not been performed or is not reported |
| **6. Follow-up period appropriate to the aim of the study** |  | ≥ 3 months | < 3 months | Not reported |
| **7. Loss to follow-up** |  | ≤ 5% | > 5% and ≤ 20% | Not reported or more than 20% |
| **8. Prospective calculation of the study size** |  | Power analysis has been performed | Explanation for the number of included patients without a power analysis | Not reported or not performed |

**Appendix 4.** Quality assessment of all included studies in the individual patient data meta-analysis using the MINORS criteria for non-comparative studies.

| **Supplementary table 4b: Quality assessment of all included studies** | | | | | | | | | | | | | | | | | | | | | | | | | |
| --- | --- | --- | --- | --- | --- | --- | --- | --- | --- | --- | --- | --- | --- | --- | --- | --- | --- | --- | --- | --- | --- | --- | --- | --- | --- |
| **Criteria** | Ali et al. 2017[1] | D’Journo et al. 2006[2] | Eroglu et al. 2009[3] | Fan et al. 2011[4] | Gubler et al. 2013[5] | Gupta et al. 2004[6] | Haveman et al. 2011[7] | Hermansson (2010)[8] | Kuppusamy et al. (2011)[9] | Law et al. 2017[10] | Linden et al. 2007[11] | Lindenmann et al. 2013[12] | Loske et al. 2015[13] | Persson et al. 2014[14] | Richardson et al. 2005[15] | Salminen et al. 2009[16] | Seven et al. 2013[17] | Soreide et al. 2012[18] | Sudarshan et al. 2016[19] | Sutcliffe et al. 2009[20] | Suzuki et al. 2016[21] | Van Heel et al. 2010[22] | Vanuytsel et al. 2012[23] | Vidarsdottir et al. 2010[24] | Zhou et al. 2015[25] |
| A clearly stated aim | 1 | 2 | 1 | 1 | 1 | 0 | 1 | 1 | 1 | 0 | 0 | 1 | 1 | 1 | 0 | 0 | 0 | 1 | 1 | 1 | 1 | 1 | 2 | 1 | 1 |
| Inclusion of consecutive patients | 2 | 1 | 1 | 1 | 1 | 2 | 1 | 2 | 2 | 1 | 2 | 1 | 1 | 2 | 1 | 1 | 2 | 1 | 2 | 1 | 2 | 1 | 2 | 2 | 2 |
| Prospective collection of data | 1 | 1 | 1 | 1 | 1 | 1 | 1 | 1 | 1 | 1 | 1 | 1 | 1 | 1 | 1 | 1 | 1 | 1 | 1 | 1 | 1 | 2 | 1 | 1 | 1 |
| Endpoints appropriate to the aim of the study | 2 | 1 | 0 | 0 | 2 | 1 | 2 | 2 | 2 | 2 | 2 | 2 | 1 | 2 | 1 | 1 | 2 | 2 | 2 | 2 | 2 | 2 | 2 | 2 | 1 |
| Unbiased assessment of the study endpoint | 0 | 0 | 0 | 0 | 0 | 0 | 0 | 0 | 0 | 0 | 0 | 0 | 0 | 0 | 0 | 0 | 0 | 0 | 0 | 0 | 0 | 0 | 0 | 0 | 0 |
| Follow-up period appropriate to the aim of the study | 1 | 2 | 0 | 2 | 2 | 2 | 2 | 2 | 2 | 2 | 2 | 2 | 2 | 2 | 0 | 2 | 2 | 1 | 1 | 2 | 2 | 2 | 2 | 2 | 1 |
| Loss to follow-up less than 5% | 0 | 0 | 0 | 0 | 0 | 0 | 0 | 0 | 0 | 0 | 0 | 0 | 0 | 0 | 0 | 0 | 0 | 0 | 0 | 0 | 2 | 0 | 0 | 2 | 0 |
| Prospective calculation of the study size | 0 | 0 | 0 | 0 | 0 | 0 | 0 | 0 | 0 | 0 | 0 | 0 | 0 | 0 | 0 | 0 | 0 | 0 | 0 | 0 | 0 | 0 | 0 | 0 | 0 |
| **Total quality score MINORS** | **7** | **7** | **3** | **5** | **7** | **6** | **7** | **8** | **8** | **6** | **7** | **7** | **6** | **8** | **3** | **5** | **7** | **6** | **7** | **8** | **10** | **8** | **9** | **10** | **6** |
| **Mean score MINORS** (±SD, range) | 6.8 (1.7, 3-10) | | | | | | | | | | | |  |  |  |  |  |  |  |  |  |  |  |  |  |
| The mean score MINORS 6.8 (out of 16) reflects an overall high risk of bias.  SD, standard deviation | | | | | | | | | | | | | | | | | | | | | | | |  |  |

**Appendix 5.** Characteristics of all excluded eligible studies.

| **Supplementary table 5: Characteristics of all excluded eligible studies (n=117)** | | | | | | | | | | | | |
| --- | --- | --- | --- | --- | --- | --- | --- | --- | --- | --- | --- | --- |
| Study, year [reference] | Country/region | Inclusion period | Sample size, n^†^ | Age, year | Gender (female) | Etiology (IEP and BS) | Location | Initial treatment | Mortality, n | LOS, d [range] | ICU, n/d^‡^ | ReI, n |
| Abbas, 2009[26] | USA | 1998-2008 | 119 | 62 | 52 (44%) | 56 (47%) | P (22%) T (51%) D (27%) | S, C | 17 (14%) | NR | NR | NR |
| Abu-Daff, 2016[27] | Canada | 1984-2012 | 127 | 68 [20-94] | 45 (35%) | 97 (76%) | P (13%) T (28%) D (51%) | S, C, E | 24 (18%) | NR | NR | NR |
| Adamek, 1997[28] | Germany | 1985- 1996 | 17 | 67 [56-81] | 10 (59%) | 9 (53%) | NR | S, C | 2 (12%) | NR | NR | NR |
| Altorjay, 1997[29] | Hungary | 1979-1994 | 86 | NR | NR | 57 (66%) | P (50%) T (30%) D (20%) | S, C | 11 (13%) | NR | NR | NR |
| Altorjay, 1998[30] | Hungary | 1985- 1994 | 27 | 53 [36-79] | 16 (59%) | 18 (67%) | T (37%) D (63%) | S | 1 (4%) | 17 [12-63] | NR | NR |
| Amir, 2004[31] | The Netherlands | 1985-2001 | 38 | 49 [1-80] | 19 (50%) | 25 (66%) | P (26%) T (74%) | S, C | 1 (3%) | 36 [6-156] | 63% | 6 (16%) |
| Attar, 1990[32] | USA | 1958- 1989 | 64 | NR | 22 (34%) | 35 (55%) | NR | S, C | 19 (30%) | NR | NR | NR |
| Ayed, 2000[33] | Kuwait | 1993- 1998 | 15 | 36 [17-53] | 7 (47%) | 8 (53%) | D (100%) | S | 1 (7%) | 13 [10-28] | NR | 3 (20%) |
| Bakken, 2010[34] | USA | 2009 | 56 | 60 [25-94] | 19 (34%) | 9 (16%) | NR | E | NR | NR | NR | NR |
| Bayram, 2015[35] | Turkey | NR | 34 | 54 [10-73] | 13 (38%) | 30 (88%) | P (9%) T (91%) | S, C, E | 9 (26%) | 13 [6-40] | NR | 8 (23%) |
| Ben-David, 2014[36] | USA | 2007- 2013 | 76 | 64 [25-87] | 26 (34%) | 76 (100%) | P (3%) T (7%) D (90%) | S, C, E | 1 (1%) | 18 [1-86] | 65% | 34% |
| Bhatia, 2011[37] | Canada | 1981- 2007 | 119 | 60 [8-92] | 35 (29%) | 100 (84%) | P (13%) T (80%) D (7%) | S, C | 22 (18%) | NR | NR | NR |
| Biancari, 2014[38] | Finland | 2000- 2013 | 194 | 65 [8-93] | 116 (60%) | 146 (75%) | NR | S, C, E | 34 (18%) | 21 [1-114] | NR | 3 (2%) |
| Bladergroen, 1986[39] | The Netherlands | 1937- 1984 | 127 | 46 [0-83] | 58 (46%) | 69 (54%) | P (29%) T (24%) D (47%) | S, C | NR | NR | NR | NR |
| Bresadola, 2008[40] | Italy | 1998- 2008 | 12 | 59 ± 12 | 4 (33%) | 12 (100%) | P (42%) T (33%) D (25%) | S, C | 3 (25%) | 34 [8-75] | NR | NR |
| Bufkin, 1996[41] | USA | 1973- 1993 | 66 | 60 ± 16 | 27 (41%) | 55 (83%) | NR | S, C | 16 (24%) | 27 ± 23 | NR | NR |
| Burnett, 1990[42] | USA | 1978-1990 | 16 | 55 ± 4 | 4 (25%) | 6 (38%) | NR | S, C, E | 6 (38%) | NR | NR | NR |
| Chao, 2005[43] | Taiwan | 1995-2002 | 28 | 44 [22-85] | 8 (28%) | 19 (68%) | P (28%) T (72%) | S, C | 3 (9%) | 53 ± 7 | NR | 4 (14%) |
| Cho, 2011[44] | Korea | 2004-2010 | 15 | 53 [39-71] | 0 (0%) | 15 (100%) | D (100%) | S, E | 1 (7%) | NR | NR | NR |
| Cho, 2008[45] | Korea | 1997-2007 | 10 | 56 [41-65] | 0 (0%) | 10 (100%) | NR | S, C | 0 (0%) | 33 [18-42] | NR | NR |
| Ciarolla, 1993[46] | USA | 1985-1991 | 89 | 48 [7-82] | 48 (54%) | 7 (8%) | NR | S, C | 0 (0%) | NR | NR | NR |
| Connelly, 2013[47] | UK | 1997-2011 | 20 | 57 [20-83] | 10 (50%) | 20 (100%) | NR | S, C | 4 (20%) | 50 [1-85] | NR | NR |
| Darrien, 2013[48] | UK | 2008-2010 | 5 | 57 [30-75] | 3 (60%) | 5 (100%) | NR | E | 1 (20%) | 70 [55-109] | NR | NR |
| Das, 2001[49] | USA | 1999-2001 | 16 | NR | NR | 16 (100%) | NR | S, C | 1 (6%) | NR | NR | NR |
| David, 2011[50] | USA | 2007-2010 | 30 | 56 | 13 (44%) | 9 (30%) | NR | S, C, E | 3 (10%) | NR | NR | NR |
| D'Chunha, 2011[51] | USA | 2005-2009 | 37 | 60 [19-83] | 11 (31%) | 6 (16%) | NR | C, E | 0 (0%) | 5 [2-33] | NR | NR |
| De Aquino, 2014[52] | Brazil | 1991-2013 | 31 | 49 [21-78] | 8 (26%) | 26 (84%) | NR | S | 7 (22,5%) | NR | NR | NR |
| Dickinson, 2016[53] | USA | 2004-2014 | 16 | 68 [27-88] | NR | 16 (100%) | NR | S, E | 2 (13%) | NR | NR | 3 (19%) |
| Dziedzic, 2016[54] | Poland | 2010-2015 | 102 | 57 [21-93] | 38 (37%) | 102 (100%) | P (12%) T (24%) D (65%) | S, C, E | 10 (10%) | NR | 13 [2-67] | 14 (14%) |
| El Hajj, 2014[55] | USA | 2003-2012 | 10 | 75 [37-87] | NR | 10 (100%) | NR | E | 1 (10%) | NR | NR | NR |
| Erdogan, 2007[56] | Turkey | 1990-2006 | 28 | 59 ± 9 | 12 (43%) | 22 (79%) | P (11%) T (89%) | S, C | 3 (11%) | 18 ± 9 | NR | NR |
| Ergolu, 2004[57] | Turkey | 1989-2002 | 36 | 54 [7-76] | 15 (42%) | 25 (70%) | P (33%) T (36%) D (31%) | S, C | 5 (14%) | 24 [7-76] | NR | 0 (0%) |
| Ferguson, 1997[58] | USA | 1980-1996 | 58 | 52 [16-84] | 22 (38%) | 52 (90%) | NR | S, C | 7 (12%) | NR | NR | NR |
| Fernandez, 1999[59] | Germany | 1983-1997 | 75 | 64 [2-90] | 31 (41,3%) | 39 (52%) | P (9%) T (57%) AL (33%) | S, C | 14 (18,7%) | NR | NR | NR |
| Fischer, 2006[60] | Germany | 1997-2004 | 14 | 58 [25-81] | 4 (29%) | 14 (100%) | NR | C, E | 2 (14%) | NR | NR | NR |
| Freeman, 2007[61] | USA | 2005-2007 | 21 | 58 ± 20 | NR | 5 (24%) | NR | E | 1 (5%) | NR | NR | NR |
| Freeman, 2007[62] | USA | 2005-2007 | 17 | 54 ± 20 | NR | 8 (47%) | NR | S, E | 0 (0%) | NR | NR | NR |
| Freeman, 2009[63] | USA | 2005-2009 | 19 | 48 ± 18 | NR | 19 (100%) | NR | S, E | 0 (0%) | 9 ± 12 | NR | NR |
| Freeman, 2012[64] | USA | 2003-2010 | 187 | NR | 87 (47%) | 90 (48%) | NR | S, E | 5 (3%) | NR | NR | NR |
| Freeman, 2015[65] | USA | 2008-2015 | 117 | 59 ± 23 | NR | 82 (70%) | NR | S. E | 2 (7%) | 8 ± 11 | NR | NR |
| Freeman, 2014[66] | USA | 2007-2014 | 29 | 64 [19-83] | NR | 22 (75%) | NR | S, E | 0 (0%) | 8 ± 11 | NR | NR |
| Freeman, 2015[67] | USA | 2009-2012 | 60 | NR | NR | 41 (68%) | NR | S, E | 3 (5%) | NR | NR | NR |
| Fry, 2007[68] | Germany | 2001-2006 | 248 | 58 [14-87] | 100 (40%) | 8 (3%) | NR | S, C, E | 1 (0%) | 14 [7-33] | NR | NR |
| Gouge, 1989[69] | USA | 1975-1988 | 18 | [31-78] | 6 (33%) | 15 (83%) | NR | S, C | 3 (17%) | NR | NR | NR |
| Graeber, 1987[70] | USA | 1960-1985 | 30 | [17-83] | 12 (40%) | 30 (100%) | NR | S, C | 4 (13%) | NR | NR | NR |
| Griffin, 2008[71] | UK | 1993-2007 | 51 | 64 [18-88] | 14 (28%) | 17 (33%) | NR | S, C | 11 (22%) | 41 [3-147] | 9 [0-75] | NR |
| Griffiths, 2009[72] | UK | 1995-2008 | 34 | 64 [23-86] | 14 (41%) | 30 (88%) | NR | S, C, E | 8 (24%) | 23 [5-98] | NR | NR |
| Hasan, 2005[73] | UK | 1994-2004 | 26 | 59 [16-92] | 14 (54%) | 16 (62%) | NR | S, C | 4 (15%) | NR | NR | NR |
| Hill, 2003[74] | New Zealand | 1993-2002 | 8 | 64 [37-80] | 1 (13%) | 8 (100%) | NR | S. C | 2 (25%) | NR | NR | NR |
| Honegger, 2017[75] | Switzerland | 2009-2015 | 202 | 61 [14-93] | 98 (49%) | 13 (7%) | NR | E | 0 (0%) | NR | NR | NR |
| Huber-Lang, 2006[76] | Germany | 2001-2004 | 17 | 63 [42-77] | 6 (35%) | 7 (42%) | P (18%) T (53%) D (29%) | S, C | 3 (18%) | 39 [16-105] | NR | NR |
| Hunt, 2000[77] | Australia | 1980-1999 | 9 | 53 [36-76] | 3 (33,3%) | 9 (100%) | NR | S, C | 0 (0%) | 14 [10-25] | NR | NR |
| Iannettoni, 1997[78] | USA | 1977-1995 | 42 | 61 [19-88] | 21 (50%) | 38 (90%) | NR | S, C | 7 (16%) | NR | NR | NR |
| Johnsson, 2005[79] | Sweden | 1998-2004 | 22 | 70 [35-88] | 8 (36%) | 6 (27%) | T (50%) D (50%) | S, E | 5 (23%) | 11 [4-23] | NR | NR |
| Jougon, 2004[80] | France | 1980-2003 | 25 | 63 [32-78] | 8 (32%) | 25 (100%) | NR | S, C | 6 (24%) | 63 [30-180] | NR | NR |
| Keeling, 2010[81] | USA | 1997-2008 | 97 | 61 ± 15,6 | 45 (46%) | 72 (74%) | NR | S, C | 8 (8%) | 25 ± 20 | NR | NR |
| Kiernan, 2003[82] | USA | 1979-2002 | 62 | 59 [20-92] | 22 (35%) | 58 (94%) | NR | S, C, E | 12 (20%) | NR | NR | NR |
| Kiernan, 2006[83] | USA | 1988-2005 | 48 | 59 [20-92] | 14 (30%) | 43 (90%) | NR | S , C, E | 6 (12,5%) | 10 [5-59] | NR | NR |
| Kim, 2016[84] | Korea | 2007-2014 | 90 | 65 [28-85] | 22 (24%) | 7 (8%) | NR | S, C, E | 0 (0%) | NR | NR | NR |
| Koivukangas, 2012[85] | Finland | 2007-2011 | 14 | 64 [47-81] | 0 (0%) | 14 (100%) | NR | C, E | 2 (14%) | 34 [16-130] | 9 [1-30] | NR |
| Kollmar, 2003[86] | Germany | 1989-2002 | 17 | [43-76] | 1 (6%) | 17 (100%) | NR | S, C | 6 (35%) | NR | NR | NR |
| Kotsis, 1997[87] | Hungary | 1981-1996 | 36 | NR | NR | 30 (83%) | NR | S, C | 7 (20%) | NR | NR | NR |
| Kotzampassakis, 2009[88] | Switzerland | 2003-2008 | 19 | 57 [41-72] | 8 (42%) | 7 (37%) | NR | S, C | 0 (0%) | 37 [12-76] | NR | NR |
| Kroepil, 2013[89] | Germany | 2003-2009 | 22 | 56 [20-94] | 9 (41%) | 16 (72%) | NR | S | 4 (18%) | 28 | NR | NR |
| Landi, 2016[90] | Spain | 2005-2013 | 12 | 59 [37-83] | 1 (8%) | 12 (100%) | NR | S, C | 3 (25%) | 33 [5-97] | 15 [5-61] | NR |
| Lawrence, 1998[91] | UK | 1986-1996 | 30 | 61 ± 16,9 | NR | 6 (20%) | P (37%) T (63%) | S, C | 3 (10%) | 27 ± 22 | 21 [15-50] | NR |
| Lazar, 2011[92] | Hungary | 1995-2008 | 15 | 59 [29-85] | 2 (13%) | 15 (100%) | T (100%) | S, C, E | 1 (7%) | 21 [15-50] | 9 [3-30] | NR |
| Liang, 2017[93] | USA | 2010-2015 | 83 | 63 [31-85] | 27 (33%) | 36 (43%) | NR | E | 0 (0%) | NR | NR | 15 (18%) |
| Lin, 2014[94] | China | 1997-2013 | 66 | 49 ± 16,2 | 19 (29%) | 21 (32%) | NR | S, C, E | 10 (15%) | NR | NR | NR |
| Merchea, 2010[95] | USA | 1996-2008 | 77 | 70 [20-95] | 46 (60%) | 27 (35%) | P (12%) T (23%) D (13%) | S, C | 13 (17%) | NR | NR | NR |
| Minnich, 2011[96] | USA | 1998-2009 | 81 | 67 [19-88] | 27 (48%) | 56 (69%) | NR | S, C, E | 3 (5%) | 11 [2-120] | NR | NR |
| Misiak, 2017[97] | Poland | 2009-2015 | 16 | 65 [24-83] | 7 (44%) | 12 (75%) | NR | S, C | 9 (56%) | NR | NR | NR |
| Mizutani, 1997[98] | Japan | 1977-1995 | 22 | 56 [40-75] | 4 (18%) | 22 (100%) | NR | S, C | 5 (23%) | NR | NR | NR |
| Muir, 2003[99] | UK | 1995-2000 | 75 | 61 [23-89] | 35 (47%) | 66 (88%) | NR | S, C, E | 12 (16%) | NR | NR | NR |
| Navaneethan, 2014[100] | USA | 2007-2012 | 20 | 73 ± 10 | 12 (60%) | 15 (75%) | NR | S, C, E | 5 (25%) | NR | NR | NR |
| Noguchi, 2017[101] | Japan | 2008-2012 | 9 | 68 ± 10 | 0 (0%) | 9 (100%) | T (44%) D (56%) | C, E | 0 (0%) | NR | NR | NR |
| Okten, 2001[102] | Turkey | 1986-1998 | 31 | 46 [11-80] | 6 (19%) | 18 (58%) | P (68%) T (32%) | S, C | 9 (29%) | 19 [6-43] | NR | 0 (0%) |
| Orive-Calzada, 2014[103] | Spain | 2004-2010 | 56 | 61 ± 15 | 20 (36%) | 29 (52%) | P (13%) T (5%) D (82%) | E | 9 (16%) | NR | NR | NR |
| Pereira-Graterol, 2006[104] | Mexico | 1993-2003 | 6 | 41 [23-67] | 5 (83%) | 6 (100%) | NR | S, C | 1 (17%) | NR | NR | NR |
| Pezetta, 2016[105] | Switzerland | 1985-2005 | 25 | 57 [47-84] | 7 (28%) | 25 (100%) | NR | S, C | 8 (32%) | NR | NR | 4 (16%) |
| Port, 2003[106] | USA | 1990-2001 | 26 | 62 [36-89] | 12 (46%) | 9 (35%) | T (100%) | S, C | 1 (4%) | NR | NR | NR |
| Prichard, 2006[107] | Ireland | 1997-2004 | 18 | 58 [28-88] | 7 (39%) | 18 (100%) | NR | S, C | 4 (22%) | NR | NR | NR |
| Reeder, 1995[108] | USA | 1980-1993 | 33 | 50 [16-82] | 10 (30%) | 28 (85%) | NR | S, C | 3 (9%) | NR | NR | NR |
| Rohatgi, 2009[109] | UK | 2000-2007 | 6 | 66 [58-76] | 2 (33%) | 6 (100%) | NR | S, C, E | 3 (50%) | 60 [32-120] | 68 [42-93] | 3 (50%) |
| Ryom, 2011[110] | Denmark | 1997-2005 | 286 | 60[1-91] | 131 (46%) | 225 (79%) | NR | S, C | 39 (21%) | 18 [1-97] | NR | 29 (16%) |
| Sabanathan, 1994[111] | UK | 1990-1993 | 6 | 50 [39-72] | 1 (17%) | 5 (83%) | NR | S, C | 0 (0%) | 12 [9-15] | NR | NR |
| Safranek, 2014[112] | Czech Republic | 2008-2012 | 16 | NR | NR | 12 (75%) | NR | S, C, E | 4 (25%) | NR | NR | NR |
| Salo, 2013[113] | Finland | NR | 83 | NR | NR | 83 (100%) | NR | S, C, E | 17 (20%) | NR | NR | NR |
| Sato, 2014[114] | Japan | 2003-2012 | 7 | 69 ± 6 | 0 (0%) | 7 (100%) | NR | S, C, E | 0 (0%) | 27 [6-90] | NR | NR |
| Sawyer, 1995[115] | USA | 1980-1993 | 31 | 61 ± 4 | 15 (48%) | 22 (71%) | NR | S, C | 5 (16%) | 26 ± 5 | NR | NR |
| Schmidt, 2010[116] | Germany | 1998-2006 | 62 | 61 [30-79] | 22 (36%) | 35 (56%) | P (14%) T (82%) D (5%) | S, C | 9 (15%) | NR | NR | NR |
| Schrosch, 2014[117] | Germany | 2006-2013 | 35 | 67 [48-84] | 14 (40%) | 7 (20%) | NR | C, E | 2 (6%) | NR | NR | NR |
| Schweigert, 2013[118] | Germany | 2000-2011 | 38 | 60 ± 14 | 9 (24%) | 38 (100%) | NR | S, C, E | 4 (11%) | 49 ± 35 | 30 ± 22 | NR |
| Schweigert, 2016[119] | Germany | 1990-2014 | 288 | 61 ± 24 | 96 (33%) | 204 (71%) | NR | S, C, E | 57 (20%) | 27 ± 34 | NR | 36 (13%) |
| Shaker, 2010[120] | UK | 200-2008 | 27 | 65 [22-87] | 9 (33%) | 27 (100%) | NR | S, C | 5 (19%) |  | 31 [13-80] | NR |
| Sulpice, 2013[121] | France | 1985-2010 | 53 | 64 ± 13 | 7 (18%) | 53 (100%) | NR | S, C | 9 (23%) | 39 [22-59] | 12 [6-32] | 16 (41%) |
| Sulpice, 2013[122] | France | 1985-2009 | 44 | 65 ± 13 | NR | 38 (86%) | NR | S, C | 6 (15%) | 41 [30-60] | 11 [6-25] | 19 (48%) |
| Sung, 2002[123] | Korea | 1986-1999 | 25 | 40 [9-60] | 5 (20%) | 18 (72%) | NR | S | 1 (5%) | 30 ± 7 | NR | NR |
| Swinnen, 2011[124] | Belgium | 1999-2008 | 88 | 52 [18-89] | 51 (58%) | 18 (21%) | NR | S, C, E | 6 (7%) | NR | NR | NR |
| Talbot, 2017[125] | Australia | 2004-2014 | 64 | NR | NR | 41 (64%) | NR | S, C, E | 1 (2%) | NR | NR | NR |
| Teh, 2007[126] | UK | 1991-2006 | 34 | 17-85 | NR | 34 (100%) | NR | S, C | 6 (18%) | NR | NR | NR |
| Thornblade, 2017[127] | USA | 2007-2014 | 659 | 49 ± 12 | 272 (41%) | 659 (100%) | NR | S, C, E | 37 (6%) | 146 [87-264] | NR | NR |
| Tilanus, 1991[128] | The Netherlands | 1972-1989 | 59 | 57 [14-81] | 20 (34%) | 40 (68%) | NR | S, C | 17 (29%) | NR | NR | NR |
| Tomaselli, 2002[129] | Austria | 1990-1999 | 38 | NR | NR | 26 (69%) | NR | S, C, E | NR | 11 [10-19] | 1 [0-7] | NR |
| Troja, 2015[130] | Germany | 2004-2012 | 39 | 66 [16-91] | 20 (51%) | 22 (57%) | P (23%) T (36%) D (41%) | S, C, E | 8 (21%) | NR | NR | NR |
| Tuebergen, 2008[131] | Germany | 1999-2006 | 32 | NR | 10 (31%) | 10 (31%) | NR | S, C, E | 5 (15,6%) | NR | NR | NR |
| Vallbohmer, 2010[132] | Germany | 1996-2008 | 44 | 67 | 14 (32%) | 37 (84%) | NR | S, C, E | 3 (7%) | 21 [2-60] | NR | NR |
| van Boeckel, 2012[133] | The Netherlands | 2007-2010 | 52 | 60 ± 14 | 20 (39%) | 17 (33%) | P (21%) T (45%) D (34%) | C, E | 5 (10%) | NR | NR | NR |
| Voermans, 2012[134] | Belgium | 2009-2010 | 39 | 67 ± 11 | 21 (58%) | 5 (13%) | NR | E | 2 (5%) | NR | NR | NR |
| Vogel, 2005[135] | USA | 1992-2004 | 47 | 59 [18-90] | 16 (34%) | 39 (83%) | NR | S, C | 2 (4%) | 26 [3-90] | NR | NR |
| Wahed, 2014[136] | UK | 2002-2012 | 96 | 70 [18-96] | NR | 89 (93%) | NR | S, C | 27 (28%) | 32 [1-108] | 6 [1-54] | NR |
| Wang, 1996[137] | USA | 1986-1994 | 18 | 66 ± 13 | 6 (33%) | 16 (89%) | NR | S, C | 3 (17%) | NR | NR | NR |
| Wang, 2009[138] | China | 1954-2006 | 18 | NR | NR | 18 (100%) | NR | S, C | 5 (28%) | 38 [7-120] | NR | NR |
| Watson, 1998[139] | USA | 1975-1996 | 104 | 44 [6-79] | 45 (43%) | 15 (14%) | NR | S, C | 2 (2%) | 17 [7-216] | NR | NR |
| Wright, 1995[140] | USA | 1979-1994 | 28 | 67 | 9 (32%) | 28 (100%) | NR | S | 4 (14%) | 23 [9-51] | NR | NR |
| Zenga, 2015[141] | USA | 1994-2004 | 28 | 53 [6-81] | 16 (57%) | 19 (68%) | NR | S, C | 0 (0%) | 13 [5-40] | NR | NR |
| Zimmerman, 2016[142] | Germany | 1986-2011 | 80 | 64 | 28 (35%) | 43 (54%) | P (13%) T (26%) D (61%) | S, C | 23 (29%) | 24,8 | NR | NR |
| **Total number of patients:** | | | **6675** |  |  | **4360 (65%)** |  |  | **757 (11%)** |  |  |  |
| ^†^ sample size numbers may represent various types of esophageal perforation (e.g. IEP, BS, traumatic, intra-operative, malignant). Percentage of patients with IEP and BS is listed elsewhere in this table. ^‡^ ICU admittance may be reported in number of patients (n, %) admitted to the ICU; or, median number of days in the ICU (d, [range] n indicates number of patients; d, days; ICU, intensive care unit; ReI, re-intervention; %, percentage; LOS, length of hospital stay; P, proximal; T, thoracic; D, distal; S, surgery; E, endoscopy; C, conservative; NR, not reported; USA, United States of America; UK, United Kingdom. | | | | | | | | | | | | |

**Appendix 6.** Characteristics and IPD of all included eligible studies

| **Supplementary table 6: Characteristics and IPD of all included studies (n=25)** | | | | | | | | | | | | | | | | |
| --- | --- | --- | --- | --- | --- | --- | --- | --- | --- | --- | --- | --- | --- | --- | --- | --- |
| General information | | | | | Demographics | | | | Initial treatment | | | | Outcomes | | | |
| Study (year) | Center,  n | Country/ region | Inclusion period | Sample size, n | Age, y, range | Gender (male) | Etiology (BS) | Location (distal) | SUR | END | CON | SUR+ END | Mortality | ICU | ReI | LOS, d, range |
| Ali (2017)[1] | 10 | North America | January 2009 – December 2013 | 113 | 62 (18-96) | 72 (64%) | 62 (55%) | 49  (43%) | 55 (49%) | 12 (11%) | 40 (35%) | 6  (5%) | 17  (15%) | 85 (78%) | 43 (38%) | 22  (1-127) |
| D’Journo (2006)[2] | 1 | France | 1989 – 2004 | 18 | 68 (44-83) | 13 (72%) | 18 (100%) | 18 (100%) | 18 (100%) | 0  (0%) | 0  (0%) | 0  (0%) | 3  (17%) | 18 (100%) | 5 (28%) | 38  (1-77) |
| Eroglu (2009)[3] | 1 | Turkey | January 1989 – May 2008 | 49 | 51 (19-76) | 32 (65%) | 3  (6%) | 13  (27%) | 32 (65%) | 5 (10%) | 11 (22%) | 1  (2%) | 6  (12%) | 49 (100%) | 16 (33%) | 19  (6-76) |
| Fan (2011)[4] | 5 | Asia | March 1993 –  June 2010 | 35 | 64 (21-83) | 25 (71%) | 0  (0%) | 7  (20%) | 2  (6%) | 0  (0%) | 33 (94%) | 0  (0%) | 0  (0%) | 1  (3%) | 3  (9%) | 1  (1-15) |
| Gubler (2013)[5] | 1 | Switzerland | January 1999 – December 2011 | 40 | 68 (35-83) | 26 (65%) | 8  (20%) | 19  (48%) | 0  (0%) | 39 (98%) | 0  (0%) | 1  (3%) | 2  (5%) | 18 (45%) | 12 (30%) | 21  (4-137) |
| Gupta (2004)[6] | 1 | India | September 1986 – December 2011 | 20 | 36 (18-60) | 7 (35%) | 2  (10%) | 7  (35%) | 18 (90%) | 1  (5%) | 1  (5%) | 0  (0%) | 2  (10%) | 12 (60%) | 11 (55%) | 14  (4-21) |
| Haveman (2011)[7] | 1 | The Netherlands | January 2002 – October 2009 | 31 | 62 (21-88) | 23 (74%) | 31 (100%) | 31 (100%) | 26 (84%) | 1  (3%) | 1  (3%) | 3 (10%) | 5  (16%) | 28 (90%) | 8 (26%) | 37  (9-165) |
| Hermansson (2010)[8] | 4 | Scandinavia | September 1970 – September 2006 | 116 | 87 (71-106) | 73 (63%) | 49 (42%) | 76  (66%) | 101 (87%) | 4  (4%) | 11 (10%) | 0  (0%) | 24  (21%) | NR | 15 (13%) | 22  (2-132) |
| Kuppusamy (2011)[9] | 1 | USA | June 1989 –  March 2009 | 100 | 66 (22-96) | 68 (68%) | 38 (38%) | 76  (76%) | 45 (45%) | 31 (31%) | 10 (10%) | 13 (13%) | 4  (4%) | 26 (59%) | 27 (51%) | 12  (1-87) |
| Law (2017)[10] | 1 | Hong Kong | January 1997 – December 2013 | 36 | 69 (30-98) | 24 (67%) | 15 (42%) | 14  (39%) | 20 (56%) | 5 (14%) | 4  (11%) | 7 (20%) | 5  (14%) | 27 (75%) | 15 (50%) | 38  (8-241) |
| Linden (2007)[11] | 1 | USA | 1989 – 2003 | 38 | 65 (36-93) | 20 (53%) | 10 (26%) | 30  (79%) | 31 (82%) | 2  (5%) | 3  (8%) | 2  (5%) | 2  (5%) | 34 (90%) | 14 (38%) | 16  (1-86) |
| Lindenmann (2013)[12] | 1 | Austria | January 2000 – December 2009 | 78 | 58 (18-92) | 55 (71%) | 15 (19%) | 52  (67%) | 53 (68%) | 7  (9%) | 17 (22%) | 1  (1%) | 10  (13%) | 56 (72%) | 20 (26%) | 16  (1-89) |
| Loske (2015)[13] | 1 | Germany | 2007 – 2014 | 10 | 63 (28-82) | 7 (70%) | 0  (0%) | 5  (50%) | 1  (10%) | 7 (70%) | 2  (20%) | 0  (0%) | 0  (0%) | 10 (100%) | 0  (0%) | 16  (7-66) |
| Persson (2014)[14] | 1 | Sweden | March 2003 – March 2013 | 41 | 66 (31-82) | 30 (73%) | 25 (61%) | 23  (62%) | 3  (7%) | 20 (49%) | 1  (2%) | 17 (42%) | 7  (17%) | 23 (61%) | 19 (50%) | 32  (2-93) |
| Richardson (2005)[15] | 1 | USA | 1985 – 2004 | 41 | 58 (30-85) | 23 (56%) | 31 (76%) | 38  (93%) | 25 (61%) | 1  (2%) | 5  (12%) | 10 (24%) | 2  (5%) | 36 (88%) | 14 (34%) | 10  (0-38) |
| Salminen (2009)[16] | 1 | Finland | January 1999 – April 2008 | 7 | 67 (51-86) | 5 (71%) | 4  (57%) | 5  (71%) | 2  (29%) | 5 (71%) | 0  (0%) | 0  (0%) | 2  (29%) | 5  (71%) | 4 (57%) | 32 (14-120) |
| Seven (2013)[17] | 1 | USA | January 1995 – January 2012 | 13 | 70 (30-88) | 9 (69%) | 4  (31%) | 12  (93%) | 0  (0%) | 8 (62%) | 0  (0%) | 5 (39%) | 0  (0%) | 2  (15%) | 2 (15%) | 13  (1-54) |
| Soreide (2012)[18] | 2 | Norway | 2000 – 2010 | 39 | 63 (18-89) | 21 (54%) | 14 (36%) | 17  (44%) | 12 (31%) | 11 (28%) | 11 (28%) | 5 (13%) | 13  (33%) | 21 (54%) | 10 (26%) | 21  (0-110) |
| Sudarshan (2016)[19] | 1 | Canada | August 2003 – January 2016 | 33 | 63 (19-90) | 24 (73%) | 16 (49%) | 23  (70%) | 22 (67%) | 0  (0%) | 11 (33%) | 0  (0%) | 2  (6%) | 23 (70%) | 11 (33%) | 18  (2-116) |
| Sutcliffe (2009)[20] | 1 | UK | 2000 – 2007 | 22 | 68 (35-85) | 15 (68%) | 22 (100%) | 22 (100%) | 14 (64%) | 1  (5%) | 7  (32%) | 0  (0%) | 7  (32%) | 20 (91%) | 8 (36%) | 48  (1-131) |
| Suzuki (2016)[21] | 2 | USA | October 2003 – September 2014 | 6 | 58 (31-72) | 4 (67%) | 2  (33%) | 6  (100%) | 0  (0%) | 4 (67%) | 0  (0%) | 2 (33%) | 1  (17%) | 6 (100%) | 4 (67%) | 30 (12-124) |
| Van Heel (2010)[22] | 1 | The Netherlands | 2001 – 2009 | 26 | 62 (18-86) | 16 (62%) | 8  (31%) | 23  (89%) | 0  (0%) | 25 (96%) | 1  (4%) | 0  (0%) | 5  (19%) | 11 (52%) | 17 (65%) | 15  (1-68) |
| Vanuytsel (2012)[23] | 1 | Belgium | January 1992 – September 2010 | 16 | 72 (44-83) | 8 (50%) | 0  (0%) | 16 (100%) | 0  (0%) | 0  (0%) | 16 (100%) | 0  (0%) | 1  (6%) | 0  (0%) | 0  (0%) | 18  (1-37) |
| Vidarsdottir (2010)[24] | 1 | Iceland | January 1980 – December 2007 | 20 | 68 (41-90) | 11 (55%) | 7  (35%) | 8  (40%) | 16 (80%) | 0  (0%) | 4  (20%) | 0  (0%) | 0  (0%) | 15 (75%) | 5 (28%) | 19  (9-83) |
| Zhou (2015)[25] | 2 | China/ South Korea | December 1990 – April 2014 | 12 | 56 (28-69) | 6 (50%) | 0  (0%) | 4  (33%) | 5  (42%) | 2 (17%) | 5  (42%) | 0  (0%) | 0  (0%) | 5  (42%) | 3 (25%) | 13  (1-129) |
| n indicates number of patients; y, year; d, days; NR, not reported; SUR, surgery; END, endoscopy; CON, conservative; ICU, intensive care unit; ReI, re-intervention; LOS, length of hospital stay; USA, United States of America; UK, United Kingdom. | | | | | | | | | | | | | | | | |

**Appendix 7.** Comparison of baseline characteristics of included and excluded patients from the primary analysis.

| **Supplementary table 7**: differences between included and excluded patients with IEP and BS | | | | | |
| --- | --- | --- | --- | --- | --- |
|  | **All patients** | **Included** |  | **Excluded** |  |
| **Variables** | (n = 960) | (n = 672) |  | (n = 288) | *p-value* |
| **Age**, years (mean ±SD) | 64 (18) | 62 (18) |  | 67 (18) | **<0.001** |
| **Gender**: male, n (%) | 617 (65) | 424 (63) |  | 193 (67) | 0.246 |
| **Etiology of perforation**, n (%) |  |  |  |  | 0.262 |
| IEP | 576 (60) | 411 (61) |  | 165 (57) | .. |
| BS | 384 (40) | 261 (39) |  | 123 (43) | .. |
| **Location perforation**, n (%) |  |  |  |  | 0.720 |
| Proximal | 362 (38) | 252 (38) |  | 110 (39) | .. |
| Distal | 594 (62) | 420 (63) |  | 174 (61) | .. |
| **Initial treatment**, n (%) |  |  |  |  |  |
| Surgery | 501 (52) | 334 (50) |  | 167 (58) | **0.019** |
| Endoscopy | 191 (20) | 150 (22) |  | 41 (14) | **0.004** |
| Conservative only | 194 (20) | 137 (20) |  | 57 (20) | 0.833 |
| Surgery and endoscopy | 73(8) | 50 (7) |  | 23 (8) | 0.770 |
| Values represent number of patients (percentage of total in column) [n (%)], mean (SD) Bold p-values indicate that differences between the groups were statistically significant. n, number of patients; SD, standard deviation; IEP, iatrogenic esophageal perforation; BS, Boerhaave’s syndrome. | | | | | |

**References**

1. Ali, J.T., et al., *Perforated esophageal intervention focus (PERF) study: a multi-center examination of contemporary treatment.* Dis Esophagus, 2017. **30**(11): p. 1-8.

2. D'Journo, X.B., et al., *Long-term observation and functional state of the esophagus after primary repair of spontaneous esophageal rupture.* Ann Thorac Surg, 2006. **81**(5): p. 1858-62.

3. Eroglu, A., et al., *Current management of esophageal perforation: 20 years experience.* Dis Esophagus, 2009. **22**(4): p. 374-80.

4. Fan, Y., et al., *Fluoroscopically guided balloon dilation of benign esophageal strictures: incidence of esophageal rupture and its management in 589 patients.* AJR Am J Roentgenol, 2011. **197**(6): p. 1481-6.

5. Gubler, C. and P. Bauerfeind, *Self-expandable stents for benign esophageal leakages and perforations: long-term single-center experience.* Scand J Gastroenterol, 2014. **49**(1): p. 23-9.

6. Gupta, N. and L. Kaman, *Personal management of 57 consecutive patients with esophageal perforation.* Am J Surg, 2004. **187**: p. 58-63.

7. Haveman, J.W., et al., *Adequate debridement and drainage of the mediastinum using open thoracotomy or video-assisted thoracoscopic surgery for Boerhaave's syndrome.* Surg Endosc, 2011. **25**(8): p. 2492-7.

8. Hermansson, M., et al., *Esophageal perforation in South of Sweden Results of surgical treatment in 125 consecutive patients.* BMC Surgery, 2010. **10**: p. 1-7.

9. Kuppusamy, M.K., et al., *Impact of endoscopic assessment and treatment on operative and non-operative management of acute oesophageal perforation.* Br J Surg, 2011. **98**(6): p. 818-24.

10. Law, T.T., et al., *Outcomes after oesophageal perforation: a retrospective cohort study of patients with different aetiologies.* Hong Kong Med J, 2017. **23**(3): p. 231-8.

11. Linden, P.A., et al., *Modified T-tube repair of delayed esophageal perforation results in a low mortality rate similar to that seen with acute perforations.* Ann Thorac Surg, 2007. **83**(3): p. 1129-33.

12. Lindenmann, J., et al., *Management of esophageal perforation in 120 consecutive patients: clinical impact of a structured treatment algorithm.* J Gastrointest Surg, 2013. **17**(6): p. 1036-43.

13. Loske, G., et al., *Iatrogenic perforation of esophagus successfully treated with Endoscopic Vacuum Therapy (EVT).* Endosc Int Open, 2015. **3**(6): p. E547-51.

14. Persson, S., et al., *Predictors for failure of stent treatment for benign esophageal perforations - a single center 10-year experience.* World J Gastroenterol, 2014. **20**: p. 10613-19.

15. Richardson, J.D., *Management of esophageal perforations: the value of aggressive surgical treatment.* Am J Surg, 2005. **190**(2): p. 161-5.

16. Salminen, P., R. Gullichsen, and S. Laine, *Use of self-expandable metal stents for the treatment of esophageal perforations and anastomotic leaks.* Surg Endosc, 2009. **23**(7): p. 1526-30.

17. Seven, G., et al., *Partially versus fully covered self-expanding metal stents for benign and malignant esophageal conditions: a single center experience.* Surg Endosc, 2013. **27**(6): p. 2185-92.

18. Søreide, J.A., et al., *Esophageal perforation: clinical patterns and outcomes from a patient cohort of Western Norway.* Dig Surg, 2012. **29**(6): p. 494-502.

19. Sudarshan, M., et al., *Management of esophageal perforation in the endoscopic era: Is operative repair still relevant?* Surgery, 2016. **160**(4): p. 1104-1110.

20. Sutcliffe, R.P., et al., *Surgical management of Boerhaave's syndrome in a tertiary oesophagogastric centre.* Ann R Coll Surg Engl, 2009. **91**(5): p. 374-80.

21. Suzuki, T., et al., *Clinical Outcomes, Efficacy, and Adverse Events in Patients Undergoing Esophageal Stent Placement for Benign Indications.* J Clin Gastroenterol, 2016. **50**: p. 373-8.

22. van Heel, N.C., et al., *Short-term esophageal stenting in the management of benign perforations.* Am J Gastroenterol, 2010. **105**(7): p. 1515-20.

23. Vanuytsel, T., et al., *Conservative management of esophageal perforations during pneumatic dilation for idiopathic esophageal achalasia.* Clin Gastroenterol Hepatol, 2012. **10**(2): p. 142-9.

24. Vidarsdottir, H., et al., *Oesophageal perforations in Iceland: a whole population study on incidence, aetiology and surgical outcome.* Thorac Cardiovasc Surg, 2010. **58**(8): p. 476-80.

25. Zhou, W.Z., et al., *Full-thickness esophageal perforation after fluoroscopic balloon dilation: incidence and management in 820 adult patients.* AJR Am J Roentgenol, 2015. **204**(5): p. 1115-9.

26. Abbas, G., et al., *Contemporaneous management of esophageal perforation.* Surgery, 2009. **146**(4): p. 749-55; discussion 755-6.

27. Abu-Daff, S., et al., *Esophagectomy in esophageal perforations: an analysis.* Dis Esophagus, 2016. **29**(1): p. 34-40.

28. Adamek, H.E., et al., *Management of Esophageal Perforations after Therapeutic Upper Gastrointestinal Endoscopy.* Scandinavian Journal of Gastroenterology, 2009. **32**(5): p. 411-414.

29. Altorjay, A., et al., *Nonoperative Management of Esophageal Perforations. Is It Justified?* Annals of Surgery, 1997. **4**: p. 415-21.

30. Altorjay, A., et al., *The Role of Esophagectomy in the Management of Esophageal Perforations.* Ann Thorac Surg, 1998. **65**: p. 1433-6.

31. Amir, A.I., H.v. Dullemen, and J.T.M. Plukker, *Selective approach in the treatment of esophageal perforations.* Scandinavian Journal of Gastroenterology, 2009. **39**(5): p. 418-422.

32. Attar, S., et al., *Esophageal Perforation: A Therapeutic Challenge.* Ann Thorac Surg, 1990. **50**(45-51).

33. Ayed, A.K., H.J. Al-Din, and S.K. Asfar, *Reinforced Primary repair of Early Distal Oesophageal Perforation.* 2000. **166**: p. 938-41.

34. Bakken, J.C., et al., *Use of a fully covered self-expandable metal stent for the treatment of benign esophageal diseases.* Gastrointest Endosc, 2010. **72**(4): p. 712-20.

35. Bayram, A.S., et al., *The success of surgery in the first 24 hours in patients with esophageal perforation.* Eurasian J Med, 2015. **47**(1): p. 41-7.

36. Ben-David, K., et al., *Esophageal perforation management using a multidisciplinary minimally invasive treatment algorithm.* J Am Coll Surg, 2014. **218**(4): p. 768-74.

37. Bhatia, P., et al., *Current concepts in the management of esophageal perforations: a twenty-seven year Canadian experience.* Ann Thorac Surg, 2011. **92**(1): p. 209-15.

38. Biancari, F., et al., *Outcome of patients with esophageal perforations: a multicenter study.* World J Surg, 2014. **38**(4): p. 902-9.

39. Bladergroen, M.R., J.E. Lowe, and R.W. Postlethwait, *Diagnosis and Recommended Management of Esophageal Perforation and Rupture.* Ann Thorac Surg, 1986. **42**: p. 235-239.

40. Bresadola, V., et al., *Treatment of perforation in the healthy esophagus: analysis of 12 cases.* Langenbecks Arch Surg, 2008. **393**(2): p. 135-40.

41. Bufkin, B.L., J.I. Miller, and K.A. Mansour, *Esophageal Perforation: Emphasis on Management.* Ann Thorac Surg, 1996. **61**: p. 1447-52.

42. Burnett, C.M., A.S. Rosemurgy, and E.A. Pfeiffer, *Life-threatening acute posterior mediastinitis due to esophageal perforation.* Ann Thorac Surg, 1990. **49**(6): p. 979-83.

43. Chao, Y.K., et al., *Treatment of esophageal perforation in a referral center in taiwan.* Surg Today, 2005. **35**(10): p. 828-32.

44. Cho, J.S., et al., *Thoracoscopic primary esophageal repair in patients with Boerhaave's syndrome.* Ann Thorac Surg, 2011. **91**(5): p. 1552-5.

45. Cho, S., et al., *Primary esophageal repair in Boerhaave's syndrome.* Dis Esophagus, 2008. **21**(7): p. 660-3.

46. Ciarolla, D.A. and M. Traube, *Achalasia. Short-term clinical monitoring after pneumatic dilation.* Dig Dis Sci, 1993. **38**(10): p. 1905-8.

47. Connelly, C.L., P.J. Lamb, and S. Paterson-Brown, *Outcomes following Boerhaave's syndrome.* Ann R Coll Surg Engl, 2013. **95**(8): p. 557-60.

48. Darrien, J.H. and H. Kasem, *Minimally invasive endoscopic therapy for the management of Boerhaave's syndrome.* Ann R Coll Surg Engl, 2013. **95**(8): p. 552-6.

49. Das, A., M.V. Sivak, and A. Chak, *Cervical esophageal perforation during EUS: A national survey.* Gastrointestinal Endoscopy, 2001. **53**(6): p. 599-602.

50. David, E.A., M.P. Kim, and S.H. Blackmon, *Esophageal salvage with removable covered self-expanding metal stents in the setting of intrathoracic esophageal leakage.* Am J Surg, 2011. **202**(6): p. 796-801; discussion 801.

51. D'Cunha, J., et al., *Esophageal stents for anastomotic leaks and perforations.* J Thorac Cardiovasc Surg, 2011. **142**(1): p. 39-46 e1.

52. de Aquino, J.L., et al., *Evaluation of urgent esophagectomy in esophageal perforation.* Arq Bras Cir Dig, 2014. **27**(4): p. 247-50.

53. Dickinson, K.J., et al., *Utility of endoscopic therapy in the management of Boerhaave syndrome.* Endosc Int Open, 2016. **4**(11): p. E1146-E1150.

54. Dziedzic, D., J. Prokopowicz, and T. Orlowski, *Open surgery versus stent placement in failed primary surgical treatment of esophageal perforation - a single institutional experience.* Scand J Gastroenterol, 2016. **51**(9): p. 1031-6.

55. El, H., II, et al., *Treatment of esophageal leaks, fistulae, and perforations with temporary stents: evaluation of efficacy, adverse events, and factors associated with successful outcomes.* Gastrointest Endosc, 2014. **79**(4): p. 589-98.

56. Erdogan, A., et al., *The sealing effect of a fibrin tissue patch on the esophageal perforation area in primary repair.* World J Surg, 2007. **31**(11): p. 2199-203.

57. Eroglu, A., et al., *Esophageal perforation: the importance of early diagnosis and primary repair.* Dis Esophagus, 2004. **17**(91-94).

58. Ferguson, M.K., L.B. Reeder, and J. Olak, *Outcome After Failed Initial Therapy for Rupture of the Esophagus or Intrathoracic Stomach.* J Gastrointest Surg, 1997. **1**: p. 34-39.

59. Fernandez, F.F., et al., *Treatment of endoscopic esophageal perforation.* Surg Endosc, 1999. **13**: p. 962-66.

60. Fischer, A., et al., *Nonoperative treatment of 15 benign esophageal perforations with self-expandable covered metal stents.* Ann Thorac Surg, 2006. **81**(2): p. 467-72.

61. Freeman, R.K., J.M. Van Woerkom, and A.J. Ascioti, *Esophageal stent placement for the treatment of iatrogenic intrathoracic esophageal perforation.* Ann Thorac Surg, 2007. **83**(6): p. 2003-7; discussion 2007-8.

62. Freeman, R.K., A.J. Ascioti, and T.C. Wozniak, *Postoperative esophageal leak management with the Polyflex esophageal stent.* J Thorac Cardiovasc Surg, 2007. **133**(2): p. 333-8.

63. Freeman, R.K., et al., *Esophageal stent placement for the treatment of spontaneous esophageal perforations.* Ann Thorac Surg, 2009. **88**(1): p. 194-8.

64. Freeman, R.K., et al., *Analysis of unsuccessful esophageal stent placements for esophageal perforation, fistula, or anastomotic leak.* Ann Thorac Surg, 2012. **94**(3): p. 959-64; discussion 964-5.

65. Freeman, R.K., et al., *An Assessment of the Optimal Time for Removal of Esophageal Stents Used in the Treatment of an Esophageal Anastomotic Leak or Perforation.* Ann Thorac Surg, 2015. **100**(2): p. 422-8.

66. Freeman, R.K., et al., *An analysis of esophageal stent placement for persistent leak after the operative repair of intrathoracic esophageal perforations.* Ann Thorac Surg, 2014. **97**(5): p. 1715-9; discussion 1719-20.

67. Freeman, R.K., et al., *A propensity-matched comparison of cost and outcomes after esophageal stent placement or primary surgical repair for iatrogenic esophageal perforation.* J Thorac Cardiovasc Surg, 2015. **149**(6): p. 1550-5.

68. Fry, L.C., et al., *Incidence, clinical management and outcomes of esophageal perforations after endoscopic dilatation.* Z Gastroenterol, 2007. **45**(11): p. 1180-4.

69. Gouge, T.H., H.J. Depan, and F.C. Spencer, *Experience with the Grillo pleural wrap procedure in 18 patients with perforation of the thoracic esophagus.* Ann Surg, 1989. **209**(5): p. 612-7.

70. Graeber, G.M., et al., *A Comparison of Patients with Endoscopic Esophageal Perforations and Patients with Boerhaave's Syndrome.* Chest, 1987. **92**(6): p. 995-998.

71. Griffin, S.M., et al., *Spontaneous rupture of the oesophagus.* Br J Surg, 2008. **95**(9): p. 1115-20.

72. Griffiths, E.A., et al., *Thirty-four cases of esophageal perforation: the experience of a district general hospital in the UK.* Dis Esophagus, 2009. **22**(7): p. 616-25.

73. Hasan, S., A.N. Jilaihawi, and D. Prakash, *Conservative management of iatrogenic oesophageal perforations--a viable option.* Eur J Cardiothorac Surg, 2005. **28**(1): p. 7-10.

74. Hill, A.G., A.T. Tiu, and I.G. Martin, *Boerhaave's syndrome: 10 years experience and review of the literature.* ANZ J Surg, 2003. **73**(1008-10).

75. Honegger, C., et al., *Establishment of Over-The-Scope-Clips (OTSC(R)) in daily endoscopic routine.* United European Gastroenterol J, 2017. **5**(2): p. 247-254.

76. Huber-Lang, M., et al., *Esophageal perforation: principles of diagnosis and surgical management.* Surg Today, 2006. **36**(4): p. 332-40.

77. Hunt, D.R., et al., *Management of Esophageal Perforation After Pneumatic Dilation for Achalasia.* J Gastrointest Surg, 2000. **4**(411-15).

78. Iannettoni, M.D., et al., *Functional Outcome After Surgical Treatment of Esophageal Perforation.* Ann Thorac Surg, 1997. **61**(1606-10).

79. Johnsson, E., L. Lundell, and B. Liedman, *Sealing of esophageal perforation or ruptures with expandable metallic stents: A prospective controlled study on treatment efficacy and limitations.* Dis Esophagus, 2005. **18**: p. 262-66.

80. Jougon, J., et al., *Primary esophageal repair for Boerhaave's syndrome whatever the free interval between perforation and treatment.* Eur J Cardiothorac Surg, 2004. **25**(4): p. 475-9.

81. Keeling, W.B., et al., *Low mortality after treatment for esophageal perforation: a single-center experience.* Ann Thorac Surg, 2010. **90**(5): p. 1669-73; discussion 1673.

82. Kiernan, P.D., et al., *Thoracic esophageal perforations.* South Med J, 2003. **96**(2): p. 18-63.

83. Kiernan, P.D., et al., *Thoracic esophageal perforation: one surgeon’s experience.* Dis Esophagus, 2006. **19**: p. 24-30.

84. Kim, H.J., et al., *Clinical outcomes of and management strategy for perforations associated with endoscopic submucosal dissection of an upper gastrointestinal epithelial neoplasm.* Surg Endosc, 2016. **30**(11): p. 5059-5067.

85. Koivukangas, V., et al., *Esophageal stenting for spontaneous esophageal perforation.* J Trauma Acute Care Surg, 2012. **73**(4): p. 1011-3.

86. Kollmar, O., et al., *Boerhaave’s Syndrome: Primary Repair vs. Esophageal Resection—Case Reports and Meta-Analysis of the Literature.* J Gastrointest Surg, 2003. **7**: p. 726-34.

87. Kotsis, L., S. Kostic, and K. Zubovits, *Multimodality Treatment of Esophageal Disruptions.* Chest, 1997. **112**(5): p. 1304-1309.

88. Kotzampassakis, N., et al., *Esophageal leaks repaired by a muscle onlay approach in the presence of mediastinal sepsis.* Ann Thorac Surg, 2009. **88**(3): p. 966-72.

89. Kroepil, F., et al., *Treatment of early and delayed esophageal perforation.* Indian J Surg, 2013. **75**(6): p. 469-72.

90. Landi, F., et al., *Treatment of Boerhaave’s Syndrome: Specialized Esophago-Gastric Unit Experience on Twelve Patients.* European Surgery, 2016. **48**(4): p. 235-240.

91. Lawrence, D.R., et al., *latrogenic oesophageal perforations: a clinical review.* Ann R Coll Surg Engl, 1998. **80**(115-8).

92. Lazar, G., Jr., et al., *A successful strategy for surgical treatment of Boerhaave's syndrome.* Surg Endosc, 2011. **25**(11): p. 3613-9.

93. Liang, D.H., et al., *Clinical outcomes following self-expanding metal stent placement for esophageal salvage.* J Thorac Cardiovasc Surg, 2017. **154**(3): p. 1145-1150.

94. Lin, Y., et al., *Management of thoracic esophageal perforation.* World J Surg, 2014. **38**(5): p. 1093-9.

95. Merchea, A., et al., *Esophagogastroduodenoscopy-associated gastrointestinal perforations: a single-center experience.* Surgery, 2010. **148**(4): p. 876-80; discussion 881-2.

96. Minnich, D.J., et al., *Management of thoracic esophageal perforations.* Eur J Cardiothorac Surg, 2011. **40**(4): p. 931-7.

97. Misiak, P., et al., *Oesophageal perforation - therapeutic and diagnostics challenge. Retrospective, single-center case report analysis (2009-2015).* Pol Przegl Chir, 2017. **89**(4): p. 1-4.

98. Mizutani, K., et al., *The Diagnosis and Treatment of Esophageal Perforations Resulting from Nonmalignant Causes.* Surg Today, 1997. **27**: p. 793-800.

99. Muir, A.D., et al., *Treatment and outcomes of oesophageal perforation in a tertiary referral centre.* Eur J Cardiothorac Surg, 2003. **23**: p. 799-804.

100. Navaneethan, U., et al., *Timing of esophageal stent placement and outcomes in patients with esophageal perforation: a single-center experience.* Surgical Endoscopy, 2014. **29**(3): p. 700-707.

101. Noguchi, M., et al., *Risk factors for intraoperative perforation during endoscopic submucosal dissection of superficial esophageal squamous cell carcinoma.* World J Gastroenterol, 2017. **23**(3): p. 478-485.

102. Okten, I., et al., *Management of Esophageal Perforation.* Surg Today, 2001. **31**: p. 36-39.

103. Orive-Calzada, A., et al., *Closure of Benign Leaks, Perforations, and Fistulas With Temporary Placement of Fully Covered Metal Stents: A Retrospective Analysis.* Surg Laparosc Endosc Percutan Tech, 2014. **24**(528-36).

104. Pereira-Graterol, F. and M. Moreno-Portillo, *Distal Esophageal Perforation Repair During Laparoscopic Esophagomyotomy: Evaluation of Outcomes and Review of Surgical Technique.* Journal of Laparoendoscopic and Avanced Surgical Techniques, 2006. **16**(6): p. 587-92.

105. Pezzetta, E., et al., *The surgical management of spontaneous esophageal perforation (Boerhaave's syndrome) 20 years of experience.* Biosci Trends, 2016. **10**(2): p. 120-4.

106. Port, J.L., et al., *Thoracic esophageal perforations: a decade of experience.* The Annals of Thoracic Surgery, 2003. **75**(4): p. 1071-1074.

107. Prichard, R., et al., *Management of spontaneous rupture of the oesophagus (Boerhaave's syndrome): Single centre experience of 18 cases.* Irish Journal of Medical Science, 2006. **175**(4): p. 66-70.

108. Reeder, L.B., V.J. DeFilippi, and M.K. Ferguson, *Current results of therapy for esophageal perforation.* Am J Surg, 1995. **169**: p. 615-7.

109. Rohatgi, A., et al., *The role of oesophageal diversion and exclusion in the management of oesophageal perforations.* Int J Surg, 2009. **7**(2): p. 142-4.

110. Ryom, P., et al., *Aetiology, treatment and mortality after oesophageal perforation in Denmark.* Dan Med Bul, 2011. **58**(5): p. 1-4.

111. Sabanathan, S., J. Eng, and J. Richardson, *Surgical management of intrathoracic oesophageal rupture.* Br J Surg, 1994. **81**: p. 863-65.

112. Safranek, J., et al., *Esophageal stents for less invasive treatment of mediastinitis.* Wideochir Inne Tech Maloinwazyjne, 2014. **9**(1): p. 1-5.

113. Salo, J., et al., *Boerhaave’s Syndrome: Lessons Learned from 83 Cases over Three Decades.* Scand J Gastroenterol, 2013. **102**: p. 271-73.

114. Sato, H., et al., *Clinical experience of esophageal perforation occurring with endoscopic submucosal dissection.* Dis Esophagus, 2014. **27**(7): p. 617-22.

115. Sawyer, R., C. Phillips, and N. Vakil, *Short- and long-term outcome of esophageal perforation.* Gastrointest Endosc, 1995. **41**(130-34).

116. Schmidt, S.C., et al., *Management of esophageal perforations.* Surg Endosc, 2010. **24**(11): p. 2809-13.

117. Schorsch, T., C. Muller, and G. Loske, *[Endoscopic vacuum therapy of perforations and anastomotic insufficiency of the esophagus].* Chirurg, 2014. **85**(12): p. 1081-93.

118. Schweigert, M., et al., *Endoscopic Stent Insertion versus Primary Operative Management for Spontaneous Rupture of the Esophagus (Boerhaave Syndrome): An International Study Comparing the Outcome.* The American Surgeon, 2013. **79**: p. 634-40.

119. Schweigert, M., et al., *Spotlight on esophageal perforation: A multinational study using the Pittsburgh esophageal perforation severity scoring system.* J Thorac Cardiovasc Surg, 2016. **151**(4): p. 1002-9.

120. Shaker, H., et al., *The influence of the 'golden 24-h rule' on the prognosis of oesophageal perforation in the modern era.* Eur J Cardiothorac Surg, 2010. **38**(2): p. 216-22.

121. Sulpice, L., et al., *Conservative surgical management of Boerhaave's syndrome: experience of two tertiary referral centers.* Int J Surg, 2013. **11**(1): p. 64-7.

122. Sulpice, L., et al., *Surgical treatment of esophageal perforations: the importance of a primary repair.* Surg Today, 2013. **43**(7): p. 727-31.

123. Sung, S.W., et al., *Surgery in thoracic esophageal perforation: primary repair is feasible.* Dis Esophagus, 2002. **15**: p. 204-9.

124. Swinnen, J., et al., *Self-expandable metal stents for the treatment of benign upper GI leaks and perforations.* Gastrointest Endosc, 2011. **73**(5): p. 890-9.

125. Talbot, M., G. Yee, and P. Saxena, *Endoscopic modalities for upper gastrointestinal leaks, fistulae and perforations.* ANZ J Surg, 2017. **87**(3): p. 171-176.

126. Teh, E., et al., *Boerhaave's syndrome: a review of management and outcome.* Interact Cardiovasc Thorac Surg, 2007. **6**(5): p. 640-3.

127. Thornblade, L.W., et al., *A Nationwide Rise in the Use of Stents for Benign Esophageal Perforation.* Ann Thorac Surg, 2017. **104**(1): p. 227-233.

128. Tilanus, H.W., et al., *Treatment of oesophageal perforation: a multivariate analysis.* Br J Surg, 1991. **78**: p. 852-85.

129. Tomaselli, F., et al., *Management of Iatrogenous Esophagus Perforation.* Thorac Cardiovasc Surg, 2002. **50**: p. 168-173.

130. Troja, A., et al., *Treatment Of Esophageal Perforation: A Single-Center Expertise.* Scand J Surg, 2014. **104**: p. 191-95.

131. Tuebergen, D., et al., *Treatment of thoracic esophageal anastomotic leaks and esophageal perforations with endoluminal stents: efficacy and current limitations.* J Gastrointest Surg, 2008. **12**(7): p. 1168-76.

132. Vallbohmer, D., et al., *Options in the management of esophageal perforation: analysis over a 12-year period.* Dis Esophagus, 2010. **23**(3): p. 185-90.

133. Van Boeckel, P.G.A., et al., *Fully covered self-expandable metal stents (SEMS), partially covered SEMS and self-expandable plastic stents for the treatment of benign esophageal ruptures and anastomotic leaks.* BMC Gastroenterol, 2012. **12**(19): p. 1-7.

134. Voermans, R.P., et al., *Efficacy of endoscopic closure of acute perforations of the gastrointestinal tract.* Clin Gastroenterol Hepatol, 2012. **10**(6): p. 603-8.

135. Vogel, S.B., et al., *Esophageal Perforation in Adults.* Annals of Surgery, 2005. **241**(6): p. 1016-1023.

136. Wahed, S., et al., *Spectrum of oesophageal perforations and their influence on management.* Br J Surg, 2014. **101**(1): p. e156-62.

137. Wang, N., et al., *Delayed primary repair of intrathoracic esophageal perforation: is it safe?* J Thorac Cardiovasc Surg, 1996. **111**(1): p. 114-21.

138. Wang, Y., et al., *Our experience on management of Boerhaave's syndrome with late presentation.* Dis Esophagus, 2009. **22**(1): p. 62-7.

139. Watson, T.J., et al., *Esophageal replacement of end-stage benign esopahgeal disease.* J Thorac Cardiovasc Surg, 1998. **115**(6): p. 1241-49.

140. Wright, C.D., et al., *Reinforced Primary Repair of Thoracic Esophageal Perforation.* Ann Thorac Surg, 1995. **60**: p. 245-49.

141. Zenga, J., et al., *Management of cervical esophageal and hypopharyngeal perforations.* Am J Otolaryngol, 2015. **36**(5): p. 678-85.

142. Zimmermann, M., et al., *Predictors of Morbidity and Mortality in Esophageal Perforation: Retrospective Study of 80 Patients.* Scand J Surg, 2017. **106**(2): p. 126-132.
